# In vivo imaging of the human eye using a 2-photon-excited fluorescence scanning laser ophthalmoscope

**DOI:** 10.1172/JCI154218

**Published:** 2022-01-18

**Authors:** Jakub Boguslawski, Grazyna Palczewska, Slawomir Tomczewski, Jadwiga Milkiewicz, Piotr Kasprzycki, Dorota Stachowiak, Katarzyna Komar, Marcin J. Marzejon, Bartosz L. Sikorski, Arkadiusz Hudzikowski, Aleksander Głuszek, Zbigniew Łaszczych, Karol Karnowski, Grzegorz Soboń, Krzysztof Palczewski, Maciej Wojtkowski

**Affiliations:** 1 International Center for Translational Eye Research and; 2 Department of Physical Chemistry of Biological Systems, Institute of Physical Chemistry, Polish Academy of Sciences, Warsaw, Poland.; 3 Laser and Fiber Electronics Group, Faculty of Electronics, Photonics and Microsystems, Wrocław University of Science and Technology, Wrocław, Poland.; 4 Department of Medical Devices, Polgenix, Inc., Cleveland, Ohio, USA.; 5 Department of Ophthalmology, Gavin Herbert Eye Institute, University of California, Irvine, California, USA.; 6 Faculty of Physics, Astronomy and Informatics, Nicolaus Copernicus University, Torun, Poland.; 7 Department of Metrology and Optoelectronics, Faculty of Electronics, Telecommunications and Informatics, Gdansk University of Technology, Gdansk, Poland.; 8 Department of Ophthalmology, Nicolaus Copernicus University, Bydgoszcz, Poland.; 9 Oculomedica Eye Research & Development Center, Bydgoszcz, Poland.; 10 Department of Physiology & Biophysics, School of Medicine,; 11 Department of Chemistry, and; 12 Department of Molecular Biology & Biochemistry, University of California, Irvine, California, USA.

**Keywords:** Ophthalmology, Medical devices

## Abstract

**Background:**

Noninvasive assessment of metabolic processes that sustain regeneration of human retinal visual pigments (visual cycle) is essential to improve ophthalmic diagnostics and to accelerate development of new treatments to counter retinal diseases. Fluorescent vitamin A derivatives, which are the chemical intermediates of these processes, are highly sensitive to UV light; thus, safe analyses of these processes in humans are currently beyond the reach of even the most modern ocular imaging modalities.

**Methods:**

We present a compact, 2-photon-excited fluorescence scanning laser ophthalmoscope and spectrally resolved images of the human retina based on 2-photon excitation (TPE) with near-infrared light. A custom Er:fiber laser with integrated pulse selection, along with intelligent postprocessing of data, enables excitation with low laser power and precise measurement of weak signals.

**Results:**

We demonstrate spectrally resolved TPE fundus images of human subjects. Comparison of TPE data between human and mouse models of retinal diseases revealed similarity with mouse models that rapidly accumulate bisretinoid condensation products. Thus, visual cycle intermediates and toxic byproducts of this metabolic pathway can be measured and quantified by TPE imaging.

**Conclusion:**

Our work establishes a TPE instrument and measurement method for noninvasive metabolic assessment of the human retina. This approach opens the possibility for monitoring eye diseases in the earliest stages before structural damage to the retina occurs.

**Funding:**

NIH, Research to Prevent Blindness, Foundation for Polish Science, European Regional Development Fund, Polish National Agency for Academic Exchange, and Polish Ministry of Science and Higher Education.

## Graphical abstract

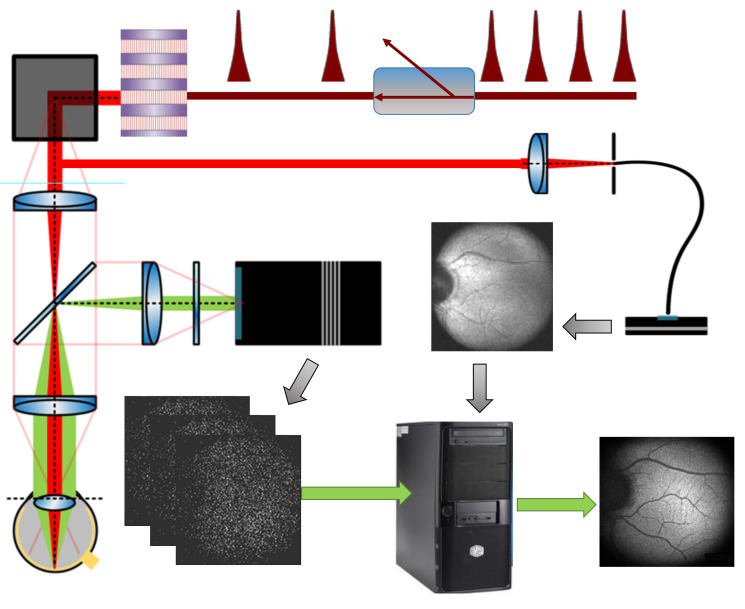

## Introduction

Ophthalmic imaging techniques are cornerstones in diagnosing retinal pathologies, disease management, and as a measure of surgical outcomes. In the last few decades, improvements in noninvasive imaging techniques have revolutionized the practice of ophthalmology. For example, optical coherence tomography (OCT) (1), scanning laser ophthalmoscopy (SLO) (2), and fundus autofluorescence (FAF) imaging (3) are imaging modalities that provide significant structural information about the back of the eye, complementary to traditional fundus photography. These 3 techniques continue to undergo further innovative transformations.

Noninvasive assessment of metabolic processes that sustain regeneration of visual pigment in the human eye is essential for the development of therapies against degenerative retinal diseases. Although existing optical imaging tools can provide high-resolution images, they reveal tissue dysfunction only when a structural phenotype already exists and, therefore, they are insensitive to early or low-grade tissue dysfunction. For example, age-related macular degeneration (AMD) is one of the most common blinding diseases affecting the world’s aging population. However, optical imaging reveals phenotypic abnormalities after disease begins, and no visual function testing can discern early disease from normal retinal function (4). Different aspects of retinal tissue function are encoded at the biochemical level through intrinsically fluorescent metabolites fundamental to visual processing and accessible by 2-photon excitation (TPE). Multiphoton-excited fluorescence imaging is an advanced high-resolution functional measurement technique that could reveal different features of the retina to further improve clinical diagnostic capabilities and accelerate the development of new treatments for blinding diseases by shortening preclinical and clinical trials. As opposed to traditional ophthalmic imaging with 1-photon fluorescence–based modalities, TPE offers access to vitamin A metabolites that sustain vision, such as retinyl esters and retinol with absorption maxima at 326 nm and 325 nm, respectively (5, 6). The eye is an ideal organ for imaging by the multiphoton excitation approach because eye tissues such as the sclera, cornea, lens, and retina are highly transparent to near-infrared (NIR) light. NIR excitation light nondestructively penetrates even cataractous lenses into retinal tissue, while maintaining a tightly focused laser beam (7). Imaging of the interface between the retina and the retinal pigment epithelium (RPE) is especially informative, because it reflects the health of the visual (retinoid) cycle and its changes in response to external stress, genetic manipulations, and treatments. Early detection of aberrant age- and/or disease-related changes in the retina and RPE is critical for developing new therapies and evaluating drug candidates for the treatment of retinal degeneration and other ocular pathologies.

Shorter-wavelength light (<400 nm) that would excite endogenous fluorophores, such as vitamin A–derived retinoids present in the retina, penetrates the front of the human eye relatively inefficiently, being largely absorbed and scattered by the structures there. Furthermore, illumination with wavelengths shorter than 400 nm can lead to irreversible changes in the transparency of the human cornea and lens (8). In mice, which have corneas/lenses that are more transparent in this spectral range (9), short-wavelength excitation of retinal chromophores can lead to photochemical degradation of retinoids (10, 11). Thus, nonlinear optical techniques based on TPE fluorescence (TPEF) are ideally suited for imaging deep into the retina because of the use of NIR wavelengths. NIR light excites retinal fluorophores, making it possible to monitor the metabolic transformation of visual cycle intermediates, including retinyl esters and retinol (12), which are indispensable for the regeneration of the visual chromophore (13). Some retinoids form condensation products, like A2E, that are highly fluorescent. These fluorescent compounds are biomarkers for potentially toxic retinoids that form during the development of retinal diseases, for example, Stargardt disease and AMD (14). Thus, changes in fluorescence intensity of retinal fluorophores that are actively involved in the visual cycle, and also condensation products that are associated with retinal degenerative diseases and aging, can be objectively correlated with retinal function at indicated retinal sites using new diagnostic approaches and devices.

With the current state of technology, i.e., imaging devices based on linear optics, it is possible to observe the distribution of condensation products on a macroscopic scale by autofluorescence imaging (15). Unfortunately, these methods only allow the observation of fluorescence excited by wavelengths above 400 nm due to safety and technology limitations; thus, visualization of vitamin A metabolites that sustain vision has not been demonstrated in humans (16). Work on FAF development has so far failed to demonstrate the ability to pinpoint fluorophores active in the visual cycle and distinguish them from passive fluorophores, such as components of lipofuscins.

TPEF occurs only around a small focal volume, and scattered or out-of-focus IR photons have insufficient power density to excite retinal chromophores. These characteristics contribute to the improved resolution and low noise of the images obtained from deep within the retinal layers. In previous studies, we presented a TPEF scanning laser ophthalmoscope (TPEF-SLO) for noninvasive imaging of the retina and RPE in mouse models of retinal diseases, underscoring the importance of temporal light shaping for the ability to safely and noninvasively identify retinal fluorophores based on their spectral and fluorescence lifetime properties (17–19). The TPEF-SLO imaging of retinal fluorophores with IR light enables monitoring of metabolic transformation in the retina. Although TPEF-SLO characterization of endogenous retinoids in the retina and RPE of mice has been achieved, noninvasive TPEF imaging of the human retina has not been realized due to concerns about safety, data interpretation, and cumbersome instrumentation (20). A key unanswered question is whether similar information could be safely derived from humans to that observed in genetically modified mice.

A TPEF instrument for safe imaging of the human retina can be built based on principles learned from TPEF-SLO imaging of mouse eyes (18). We present, for the first time t our knowledge, TPEF images of the living human eye, measured in 2 healthy subjects with an average light power of only 0.3 mW. In addition, we show the results of spectral analysis of the fluorescence signals measured in vivo in humans and compare them to an analogous analysis performed in rodents. This method enables the comparison of the relative content of fluorophores participating in the regeneration of the visual chromophore and those that continuously accumulate in the retina in humans. Lastly, we demonstrate no effect of TPEF-SLO exposures, sufficient to collect 120 frames, on the retinal structure by using blue-induced FAF (B-FAF), NIR-FAF, and optical coherence tomography (OCT). Furthermore, there were no effects on retinal function as measured with 1- and 2-photon perimetry.

## Results

### TPEF-SLO.

**Figure 1:**
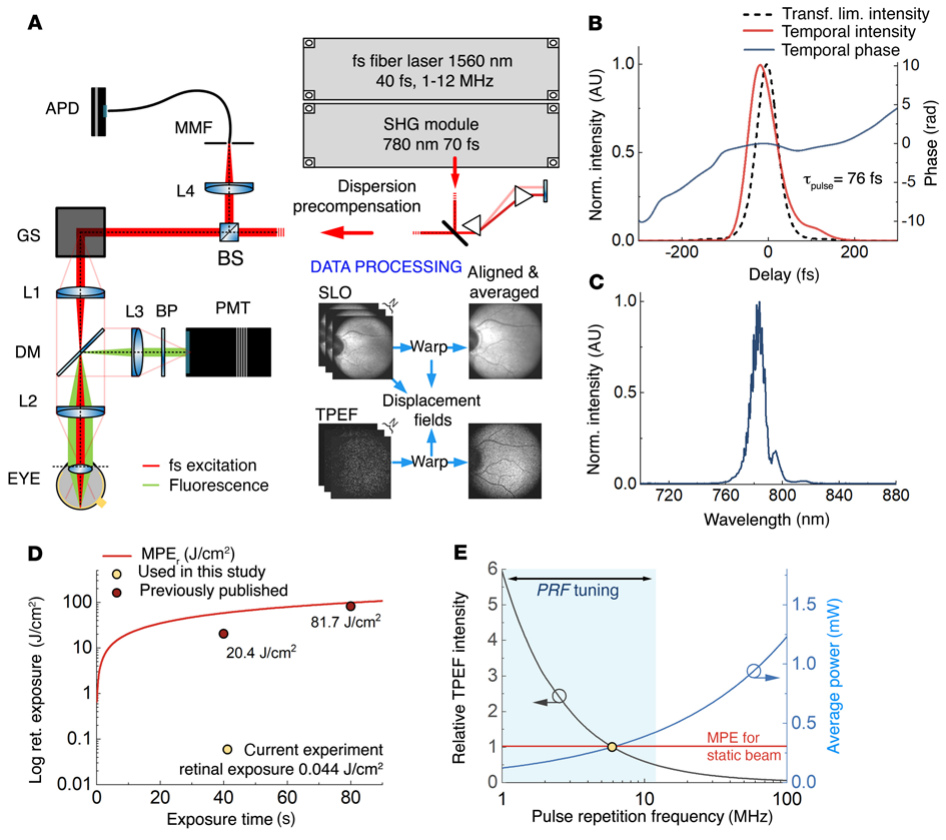
Two-photon-excited fluorescence scanning laser ophthalmoscope (TPEF-SLO) driven by a femtosecond fiber laser. (**A**) Experimental setup of TPEF-SLO, including 4 major units: femtosecond laser, second harmonic generation (SHG) module, dispersion precompensation, and SLO module; inset represents image processing (each unit is described in detail in Methods). L, lens; GS, galvanometer-based *x*-*y* scanners; DM, dichroic mirror; BP, set of bandpass filters; PMT, photomultiplier tube; MMF, multimode fiber; APD, avalanche photodiode. (**B**) Retrieved pulse intensity and phase measured in the retinal plane. (**C**) Optical spectrum of the laser measured in the pupil plane. (**D**) Retinal exposure vs. exposure time (red curve = equivalent of MPE calculated for static beam case) and comparison of retinal exposures used in this study and in Schwarz et al. (24); adapted from Schwarz et al. (24) with with permission from The Optical Society of America. (**E**) Relative TPEF intensity as a function of pulse repetition frequency (PRF, black curve), illustrating the effect of reduced PRF. Shown is the calculated average excitation power (blue curve) needed to obtain the same fluorescence intensity as for 0.3 mW and 6 MHz used in this study. Red line shows MPE calculated for static beam case and 40-second exposure time.

The experimental setup of TPEF-SLO is schematically depicted in Figure 1A, and in greater detail in the Supplemental Information and Supplemental Figure 1; supplemental material available online with this article; https://doi.org/10.1172/JCI154218DS1 We used a custom-made Er:fiber laser, providing 40-fs pulses at the central wavelength of 1560 nm. The laser was equipped with an internal pulse picker unit, allowing us to set the pulse repetition frequency (PRF) at 6 MHz within the 1–12 MHz range (21). The light beam from the fiber laser was fed to a second harmonic generation (SHG) module for frequency doubling. As a result, 70-fs pulses at the central wavelength of 780 nm were generated, suitable for TPE of endogenous retinal fluorophores. The SHG module produced nearly transform-limited pulses (22) without side lobes (Supplemental Figure 2), which is essential for maximizing the efficiency of TPE. Light (780 nm) was then guided through a prism pair compressor to precompensate for the chromatic dispersion by subsequent optical elements and the eye itself (see Methods). As a result, 76-fs pulses with close to transform-limited temporal shape were delivered to the retinal plane (Figure 1B). The corresponding optical spectrum measured in the pupil plane is depicted in Figure 1C. After dispersion precompensation, the beam entered a TPEF-SLO with *x*-*y* galvanometer scanners (GSs) and a telescope relaying the GS’s plane to the pupil plane of the eye. The TPEF-SLO allows for simultaneous frame registration in 2 channels, non-descanned fluorescence, and descanned reflectance. Reflectance images were obtained with the same light as for TPEF images and served to adjust eye position before imaging and as guidance for subsequent alignment of fluorescence frames, and to correct motion artifacts within the frame. During imaging sessions, eye movement inevitably occurs, not only between the acquisition of subsequent frames but also within the frame. Typically, a single fluorescence frame does not provide enough information for alignment; thus, reflectance images with a much higher signal-to-noise ratio (SNR) were used to calculate correction shifts for each pixel. The obtained shifts were applied to fluorescence images; we typically averaged 70–120 frames to improve the SNR. More details on the measurement procedure and image processing are in Methods and the Supplemental Information.

### Safety assessment and signal enhancement strategy.

The use of high-energy pulses in TPEF imaging previously raised concerns about the possibility of inducing tissue damage by nonlinear effects. Furthermore, exposure to wavelengths of 700 to 800 nm was shown to decrease NIR-FAF (attributed to melanin; refs. 23, 24).

The first concern has been addressed comprehensively in Palczewska et al. (18). The strategy of enhancing the 2-photon signal by lowering the PRF of the laser proved to be efficient for mouse eye imaging. Numerical modeling of the photodamage excluded the possibility of plasma formation, and thermal effects related to the presence of melanin were mitigated by imaging with a lower PRF of the laser light (18).

The observed reduction in NIR-FAF was associated with high retinal exposures and dense scanning patterns typically used in adaptive optics SLO beams (23, 24). For example, in Schwarz et al. (24), the 40-second illumination of the macaque eye with a beam of relatively low average power (0.5 mW) led to an exposure value of 20.4 J/cm^2^ because the energy of photons was concentrated on a tiny retinal area (1.3° × 1.1°). In our experiments, 40-second illumination of the retina with a 0.3-mW beam led to an exposure value of 0.044 J/cm^2^ since we illuminated a retinal area of 17.6° × 17.6°. Figure 1D compares those retinal exposures with calculated retinal maximum permissible exposure (MPE) for the static beam case (see Supplemental Information). Furthermore, the scanning pattern used in these previous studies was dense; the 71 consecutive lines overlapped in an area *tmin*, which is the minimal spot of thermal impact of the beam, assumed in American National Standards Institute (ANSI) standards (25). In the case of our system, only one-quarter of the width of the next line overlapped the previous one. The frame repetition rates used in the referenced works were also much higher than in our system. Therefore, the laser beam spent relatively more time in the same area of the retina (in terms of heat dissipation): *teff* was equal to 369 ms for 20.4 J/cm^2^ exposure in Schwarz et al. (24) and only 0.31 ms in our experiments: *teff* = *n* × *m* × *t_min_,* where *n* is the number of frames in a single 40-second measurement, *m* is the number of overlapping lines, and *tmin* is time, provided by ANSI (25), during which heat transfer from the exposed site is sufficiently small to assume that all energy delivered in this period of time was supplied in the form of 1 pulse (see Supplemental Information).

Our strategy to perform safe 2-photon imaging of the human retina was to choose the most conservative approach. In our instrument, we used the same light for generating reflectance and TPEF images. We substantially reduced the density of the scanning pattern to decrease the exposure as much as possible, and we kept the average power equal to 0.3 mW, below the safety limit for a static beam for a single 40-second measurement, i.e., 0.398 mW (see Supplemental Information). Additionally, based on 3 different approaches, we provide a safety assessment of our TPEF-SLO protocol to show that, for a constant and large scanning area (17.6° × 17.6°), the safety limit can be increased to 14.1 mW. To compare with a case of adaptive optics SLO, we also calculated limits for the smaller retinal area (1.5° × 1.5°), close to the isoplanatic patch of the eye (26) (see Supplemental Information), and it was equal to 6.9 mW. Most existing SLOs do not fulfill the limits for a static beam (27). Such a restrictive limitation on the power of the excitation beam required special means to enhance the signal of the detected TPEF.

For the same average laser power, an increase in TPEF intensity is possible via manipulation of the temporal properties of the pulse train. It was previously shown that reducing PRF while keeping other parameters fixed (average power, pulse duration; refs. 18, 21, 28) or reducing pulse duration (19) increases the TPEF signal because of the nonlinear dependence on pulse peak power. Figure 1E shows the calculated TPEF intensity at 6 MHz PRF, which we used in this work. The blue curve demonstrates the average power that would be needed to obtain the same TPEF intensity as 0.3 mW at 6 MHz for other PRFs. Thus, the reduction in PRF allowed us to register informative images with excitation power as low as 0.3 mW. We expect that further reduction in PRF will increase TPEF intensity in the human eye, which is demonstrated in the in vivo mouse eye imaging (see Supplemental Information); however, it may raise concerns about safety issues arising from nonlinear excitation.

### In vivo human eye imaging.

**Figure 2:**
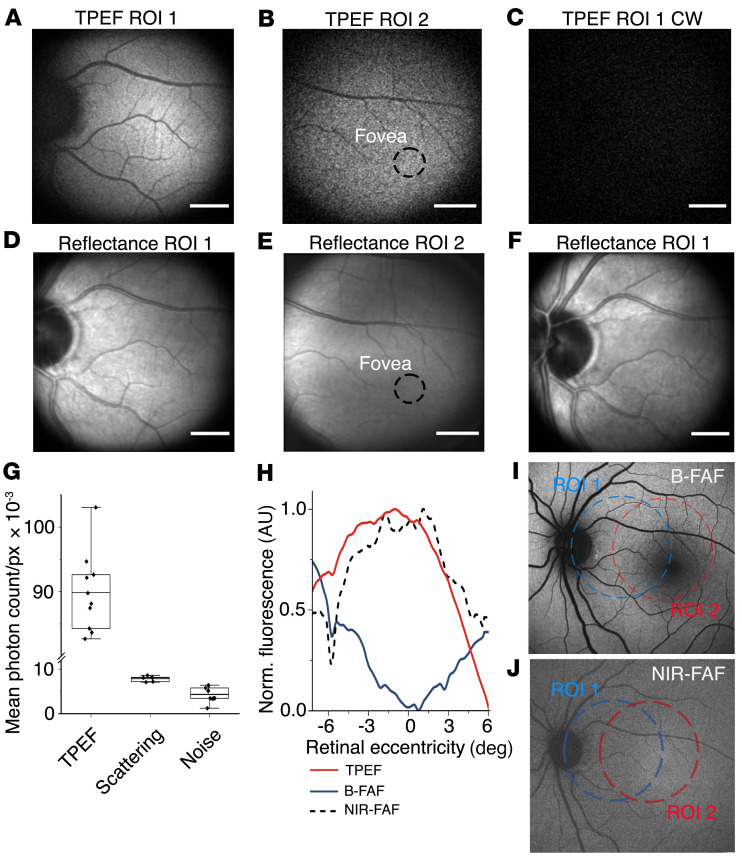
TPEF-SLO enables imaging of endogenous fundus chromophores in eye of a healthy subject 1. (**A**) TPEF-SLO image of the fundus centered at 7.7° eccentricity nasally from foveal region of interest (ROI) 1. Image obtained by averaging of 1000 frames in spectral window 400 to 700 nm. (**B**) TPEF-SLO image of the fundus centered at 2.5° eccentricity nasally from fovea, ROI 2. Image obtained by averaging 100 frames. (**C**) TPEF-SLO image from ROI 1 location acquired with continuous wave (CW) excitation at 825 nm, showing significantly decreased TPEF signal intensity. Image obtained by averaging 100 frames. (**D**–**F**) Confocal reflectance images corresponding to panels **A**–**C**. (**G**) Quantification of fluorescence intensity with respect to leakage of backscattered light and noise floor (dark counts and stray light) (*n =* 10, 5, and 10, respectively). The upper and lower bands indicate second and third quartiles, respectively; the line within the box indicates the mean value; whiskers extend to minimum and maximum values. (**H**) Normalized fluorescence as a function of eccentricity at ROI 2 compared with the corresponding region measured by B-FAF and NIR-FAF methods. (**I**) Confocal B-FAF image (488 nm excitation). (**J**) Confocal NIR-FAF image. In **I** and **J**, blue circles mark the location at 7.3° eccentricity nasally from fovea (ROI 1), and red circles mark the macular region (ROI 2). In **B** and **E**, circles outlined in dashed black dashed lines mark the location of fovea centralis. Scale bars: 1 mm.

Using TPEF-SLO, we registered representative fundus images at 2 regions of interest (ROI 1 and 2) in the fluorescence (TPEF, Figure 2, A–C) and reflectance channels (Figure 2, D–F). With TPE, the strongest fluorescence signal originates from the photoreceptor and RPE layers (29); thus, the focus was set on these layers to obtain maximum fluorescence. The TPEF images were recorded within a spectral bandwidth of 400 to 700 nm. One image frame consisted of 256 × 256 pixels with an exposure time of 20 μs per pixel. Generating TPEF-SLO images required averaging data from multiple frames. A single measurement consisted of collecting 30 frames. During one imaging session, 4 measurements, each lasting 40 seconds, were made. Consecutive measurements were separated by at least a 60-second break (details in Methods). To obtain high image quality in ROI 1 (Figure 2A), we averaged 1000 frames derived from multiple sessions performed over 3 weeks. The image recorded at 7.7° eccentricity nasally from the fovea, ROI 1, contains a reconstruction of a significant portion of the optic nerve disc and terminates in the foveal region on the temporal side. The ROI 2 area has a center at approximately 2.5° eccentricity and shows the macular area with the fovea centralis marked by a dashed black line circle (Figure 2B). Given that the full width half maximum (FWHM) of the square of the axial illumination point spread function is equal to 130 μm (Supplemental Figure 3), we attributed the observed signal to endogenous chromophores in the photoreceptor layer and RPE. In both cases, the images show no abnormalities.

To further investigate the origin of the signal recorded in the fluorescence channel, we also used 825 nm continuous wave (CW) excitation instead of femtosecond pulses (Figure 2C). All other parameters were held constant, including an average power of 0.3 mW. TPEF imaging with CW excitation showed a nearly 11.5-fold signal reduction in the fluorescence channel and no fundus features were detected. The corresponding image registered in the reflectance channel confirmed proper eye positioning and imaging location on the retina (Figure 2F). Figure 2G compares signal intensities registered in the fluorescence channel with femtosecond and CW excitations (designated backscattered light). The signal values are expressed as mean number of photon counts per pixel (pc/px) within the field of view (FOV) of the instrument. With femtosecond excitation, the average signal was equal to 89.9 × 10^3^ pc/px (SD = 5.8 × 10^3^), whereas with CW excitation, it was 7.8 × 10^3^ pc/px (SD = 6 × 10^4^). Because no TPEF signal is expected with CW excitation at this excitation power, the registered signal can be attributed to stray light, dark counts of the photomultiplier tube (PMT), and leakage of backscattered excitation photons. For the latter, as there is no confocality in the system, each part of the eye can contribute to the registered signal (e.g., the cornea or any other highly scattering part of the eye). We also compared those numbers to the instrument’s noise floor registered with a blocked laser beam; i.e., stray light and dark counts, which were equal to 4.4 × 10^3^ pc/px (SD = 1.6 × 10^3^).

### Comparison with B-FAF and NIR-FAF.

When TPEF images were compared with other autofluorescence-based eye-imaging techniques (Figure 2, H–J), many similarities in the morphology of the reconstructed structures were seen. One of the distinctive features of TPEF images is the absence of a hypofluorescent area around the fovea; in our case, this area is located in ROI 2 and can be seen in Figure 2C. To analyze this feature further, Figure 2H shows the mean pixel intensity for each row within an image as the function of eccentricity within ROI 2 for the 3 imaging methods. The pixel’s intensities were normalized to maximum intensity within the analyzed FOV for each imaging method separately. There was no decreased fluorescence area around 0° in TPEF (red curve) in contrast to B-FAF (488 nm excitation, blue curve, full image shown in Figure 2I). This can be explained by the absorption spectra of macular pigments, which absorb the excitation beam in B-FAF (30), while TPE in the NIR allowed us to bypass this effect. NIR-FAF (dashed black line, full image shown in Figure 2J) shows a distribution of fluorescence intensity similar to that of TPEF. In NIR-FAF, the excitation wavelength also bypasses the absorption of macular pigments, but the signal originates primarily from melanin (31).

### Spectral properties of TPEF of human fundus.

**Figure 3:**
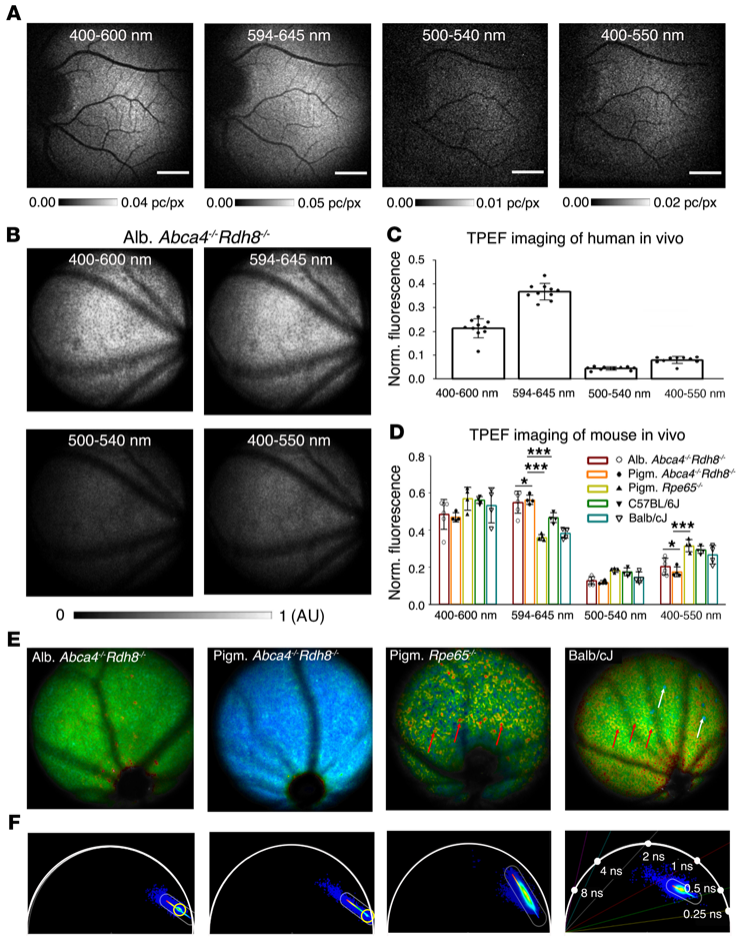
Spectral properties of TPEF of human fundus compared with selected mouse models. (**A**) Human TPEF images of subject 1 at approximately 7.5° eccentricity nasally from fovea (ROI 1), recorded in spectral ranges of 594–646 nm, 400–600 nm, 500–540 nm, and 400–550 nm, and normalized to the image acquired for 400–700 nm; 1000 frames were used. (**B**) TPEF fundus images of albino (Alb.) *Abca4^–/–^ Rdh8–/–* mice in vivo recorded in corresponding spectral ranges normalized to the TPEF image obtained in the 400–700 nm spectral range. (**C**) Plot showing relative fluorescence change in 4 spectral ranges normalized with respect to 400–700 nm for human TPEF imaging (*n =* 10). (**D**) Plot showing relative fluorescence change in 4 spectral ranges normalized with respect to 400–700 nm for 5 mouse models (*n =* 6, 4, 4, 4, and 4). Pigm., pigmented. *P > 0.2, ***P < 0.005 by 1-way ANOVA with Bonferroni’s post hoc test. (**E**) FLIM images of albino *Abca4^–/–^ Rdh8–/–*, pigmented *Abca4^–/–^ Rdh8–/–*, pigmented *Rpe65–/–*, and BALB/cJ mice. Red arrows point to retinosomes (57), and white arrows point to macrophages. (**F**) Phasor plots corresponding to data presented in panel **E**. In each universal semicircle, clusters of phasor points are color coded from blue to red, where red represents highest phasor point density. Color bars drawn through clusters of phasor points represent color scales for FLIM images in **E**. Yellow circles outline grouping of phasor points in albino and pigmented mouse RPE. Error bars represent SD.

Although TPEF images provide similar structural information to that of B-FAF and NIR-FAF, different fluorophores can be excited because of substantially altered excitation wavelengths. Retinal fluorophores can be differentiated by fluorescence lifetime and fluorescence spectrum (32). However, whenever single-photon-excited fluorescence is recorded, it does not include a contribution from endogenous fluorophores participating in the visual cycle. In TPEF imaging, this information becomes accessible. To demonstrate the differences between the fluorophores involved in the formation of retinal TPEF images, a spectral analysis of the fluorescence signals coming from the eye is required. To this end, we performed imaging in 5 different spectral windows and compared the results obtained in humans with those from measurements in animal models. To minimize the influence of systematic errors, we performed a relative comparison of the fluorescence values measured in 4 spectral windows (400–600 nm, 594–646 nm, 500–540 nm, and 400–550 nm) with the fluorescence signal measured in the entire available spectral window (400–700 nm). In these experiments, the collection of the signal was in small spectral windows; thus, the contribution of the dark noise was stronger. Ten separate imaging sessions were performed, each containing an entire set of spectral filters. Figure 3A shows that the images recorded in each spectral range varied significantly in signal intensity (note that the intensity scale in each case is adjusted for image clarity). Distinctive fundus features are visible in each image, suggesting that fluorescence is emitted over a broad spectral band extending from 400 to 700 nm. Most of the fluorescence signal was in the spectral range above 550 nm. The results presented here were obtained by aligning and averaging between 1171 and 1400 frames, depending on the emission filter used.

Evaluation of the fluorophores responsible for the results obtained in humans requires comparison with spectrally resolved TPEF images of the retina in mouse models (Figure 3, B–D). To determine which chemical intermediates are responsible for TPE-SLO signals in humans, we used the same spectral imaging conditions, namely a fluorescence excitation wavelength of 780 nm and spectral detection windows for imaging pigmented and albino wild-type (WT) mouse models and pigmented and albino mouse models of Leber congenital amaurosis and Stargardt disease, as those used in human imaging. We selected 3 types of mice according to their characteristics: (a) albino and pigmented *Abca4–/–* *Rdh8–/–* mice that accumulate an excess of retinal condensation products in the RPE; (b) pigmented *Rpe65−/−* mice that accumulate an excess of retinyl esters in the RPE and lack retinal condensation products; and (c) pigmented WT, C57BL/6J, and albino BALB/cJ mice with faster retinoid cycles (33). For each mouse, fundus images were recorded in the spectral bands corresponding to human imaging. Figure 3, C and D compare the average fluorescence intensity in each spectral band recorded in human and mouse in vivo. In both cases, the fluorescence intensities were normalized to the intensity over the entire spectral range, i.e., 400 to 700 nm, showing how much fluorescence was contained in a given spectral channel. By comparing data from albino and pigmented WT and retinal disease mouse models, we were able to assess the impact of melanin on the TPEF image. Moreover, we measured spectral differences between WT mice (either albino or pigmented) and mice that accumulated an excess of retinal condensation products (either albino or pigmented). These differences were most pronounced in the spectral bandwidths 594 to 646 nm, corresponding to retinal condensation products; and 400 to 550 nm, corresponding to retinyl esters. In the human eye, fluorescence in the 594 to 646 nm spectral range was 3.2-fold higher than in the 400 to 550 nm range. Similar results were observed in both pigmented and albino *Abca4–/–* *Rdh8–/–* mice, pointing to retinal condensation products as the main source of fluorescence in the 594 to 646 nm spectral range. Furthermore, results in pigmented and albino *Abca4–/–* *Rdh8–/–* mice were not significantly different, suggesting that melanin did not contribute substantially to the emission spectrum. In the 400 to 550 nm spectral range, fluorescence is the highest from the RPE of pigmented *Rpe65−/−*, C57BL/6J, and BALB/cJ mice, consistent with differences in the retinyl ester content of their RPE (17). These results show that the 780 nm TPEF emission spectrum of the human fundus in vivo is most similar to that of the *Abca4–/–* *Rdh8–/–* mice, indicative of the accumulation of retinal condensation products such as A2E. We also registered phasor fluorescence lifetime images (FLIMs) from mice with similar emission spectra to that of a 45-year-old human, i.e., pigmented and albino *Abca4–/–* *Rdh8–/–* mice, and mice with differing spectra, i.e., pigmented *Rpe65−/−* and BALB/cJ mice (Figure 3, E and F). As expected, phasor points from pigmented and albino *Abca4–/–* *Rdh8–/–* mice were located in the vicinity of each other, with slightly shorter fluorescence lifetimes in pigmented mice, consistent with melanin’s contribution to phasor FLIM (19). Phasor points from BALB/cJ and pigmented *Rpe65−/−* mice were shifted toward longer lifetimes, consistent with a higher contribution of retinyl esters to the RPE fluorescence (18).

### Studies in volunteers.

**Figure 4:**
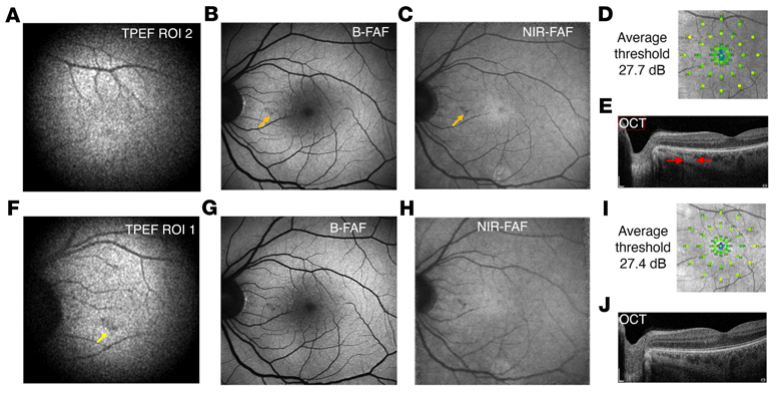
TPEF provides safe fundus imaging without any structural or functional changes. (**A**) TPEF fundus image of subject 2 centered at the fovea. Total exposure: 163 seconds. (**B**–**E**) Data obtained before TPEF-SLO imaging; yellow arrows point to depigmentation region in all. (**B**) B-FAF image. (**C**) NIR-FAF image. (**D**) Visual field tested by perimetry. (**E**) OCT b-scan; red arrows indicate the spread of the depigmentation. (**F**) TPEF fundus image of subject 2 centered at 6.9° eccentricity nasally from the fovea; the yellow arrow points to an area with a clear hypofluorescent lesion. Total exposure: 162 seconds. (**G**–**J**) Data obtained 1 month after TPEF imaging. (**G**) B-FAF. (**H**) NIR-FAF. (**I**) OCT. (**J**) Perimetry. In **A** and **F**, 100 frames were averaged to generate the images.

Evaluation of the safety of TPEF imaging required an extensive set of clinical studies. Structural (B-FAF, NIR-FAF, and OCT) as well as functional (1- and 2-photon perimetry) studies were used for this purpose. In our case, we tested the eye of a 44-year-old subject 2 weeks before and 4 weeks after exposure to the laser beam in our TPEF-SLO system. TPEF imaging was performed at 2 retinal locations. Figure 4A shows a TPEF image centered at the fovea (ROI 2), showing the macular region. The region coincides with the FOV of B-FAF and NIR-FAF, as well as perimetry measurements (Figure 4, B–E). The imaging session consisted of six 40-second exposures to the TPEF-SLO scanning laser beam at ROI 2 and six 40-second exposures at ROI 1 (Figure 4F). Each exposure was followed by at least a 60-second delay before the next.

Figure 4 compares B-FAF, NIR-FAF, and OCT images as well as photopic microperimetry results, before (panels B–E) and 1 month after the first TPEF-SLO imaging session (panels G–J). Autofluorescence examinations show typical results with no pathologies prior to and after the TPEF imaging, except for a hypofluorescent lesion visible in both B-FAF and NIR-FAF images obtained before and after TPEF-SLO imaging. This anomaly is indicated with yellow arrows in panels B, C, and F. The localization of a hypofluorescent spot in TPEF images correlates perfectly with the corresponding localization of a lesion in B-FAF and NIR-FAF. A cross-sectional OCT image of the lesion (Figure 4E) shows deeper light penetration into the choroid — red arrows outline the hypofluorescent lesion. The results of clinical autofluorescence imaging conducted 1 month after the TPEF showed no change. The visual field test done by the clinical instrument (MAIA, photopic conditions) before TPEF shows typical visual sensitivity with an average threshold of 27.7 dB, and a value of 27.4 dB 1 month after TPEF imaging. We also performed perimetry in photopic and scotopic conditions, using both 1-photon and 2-photon isomerization of visual pigments (7, 34, 35). Small changes in visual sensitivity with aging were reported earlier in scotopic perimetry testing (36); therefore, we decided to use it as another test of retinal function. Two-photon perimetry is a novel method to assess retinal function based on the nonlinear activation of visual pigments by an NIR pulsed laser beam. It was previously shown that quadratic dependence on stimulus intensity makes this method more accurate than the classical version of visual field testing (7). Using both visible and IR light, we measured the visual sensitivity thresholds at 17 different locations across the retina, at 3 eccentricities (0°, 2.5°, and 5°). Detailed results are shown in Supplemental Figures 4 and 5. For 1-photon isomerization, the average threshold before and after was 34.8 ± 7.7 dB and 36.4 ± 5.1 dB, respectively, which shows no changes in the visual field sensitivity after the TPEF imaging. Similarly, we did not observe a statistically significant difference in visibility thresholds before and after exposure to the femtosecond laser pulse for the 2-photon version of perimetry. Specifically, the thresholds were 14.3 ± 4.7 dB and 16.1 ± 4.2 dB before and after the TPEF imaging, respectively.

## Discussion

The ability to evaluate functioning of the retinoid cycle (13, 37) has become even more needed now, with the development of novel retinal therapies based on gene transfer, genome editing, and transplantation of human embryonic stem cell–derived retinal tissue layers or retinal organoids (38, 39).

In this report, we demonstrate a TPEF-SLO instrument and method that allow the measurement and spectral characterization of TPEF in the human eye in vivo. To obtain informative images and reduce the total light exposure required for TPEF imaging, we have included a fiber-based laser with integrated PRF modulation in our new instrument to increase TPE efficiency; dispersion compensation; highly efficient coupling of light into the human eye, ensuring efficient collection of fluorescent photons; spectral detection; and intelligent data postprocessing to obtain informative images with light exposure an order of magnitude below safety limits calculated for scanning beam. To achieve imaging with a larger FOV, we reduced the size of the beam on the cornea to minimize impact of optical aberrations, which in turn resulted in 21-μm lateral and 130-μm axial resolution (40). We have built a compact portable instrument using a custom-made, perfectly constant-phase light source and integrated TPEF system with a collinear reflectance channel for frame alignment, compensating for eye motion. These advances have made it possible to measure weak fluorescence signals resulting from exposure to excitation light with an average power of only 0.3 mW.

The 2-photon imaging of the retina in humans presented here arises from a quest to characterize in vivo endogenous molecular components in the retina and RPE such as retinyl esters (41) that sustain our vision, and fluorophores such as A2E (42), which aberrantly accumulate in some retinal diseases such Stargardt and AMD. Previously, fluorescence signals from retinosomes (12) in *Lrat–/–* mice, which lack retinoids in their RPE because of a genetic defect, were detected by 2-photon imaging, after transplantation of the RPE obtained from human induced pluripotent stem cells (43). However, advancement of 2-photon imaging to the human retina in vivo has been stymied by concerns about the safety of exposing the retina to pulsed IR light, uncertainty about interpretation of the data, and the difficulty of introducing large-footprint imaging equipment into the clinical environment, including the Ti:sapphire laser and associated high-upkeep instrumentation.

Recently, experiments in mice and nonlinear modeling revealed that TPE efficiency could be safely increased, and average laser power reduced, by modulating temporal properties of the light, namely PRF and pulse duration (18, 19).

Thus, we introduced a compact 2-photon imaging system with a custom-made IR light source — SHG Er:fiber laser with integrated PRF regulation. Integration of this light source enabled TPEF imaging with average optical powers 47 times below safety limits and 463 times less than exposures previously shown to produce reduction in IR autofluorescence (24). Furthermore, we compared spectrally resolved TPEF images from human and mouse retinas, showing the dominance of retinal condensation products in spectral properties in humans.

The guiding principles for the design of the compact TPEF-SLO, which facilitated successful use with humans, included (a) replacing a Ti:sapphire laser source with an easy-to-use alternative with a small footprint; (b) keeping the number of elements as few as possible, allowing for a compact, yet efficient, dispersion compensation; (c) using the same light source for both reflectance and TPEF imaging; and (d) enabling registration of weak fluorescence signals by including sensitive photon-counting detection and advanced data postprocessing.

The most challenging factor in development of the compact instrument was the replacement of femtosecond light source, a Ti:sapphire laser. This kind of laser is a large-footprint device that requires an optical table, active water cooler, and driving electronics. To overcome this obstacle, we have developed a custom, compact fiber-based laser with integrated pulse selection unit, that has a size of 415 × 120 × 280 mm (width × height × length). The laser’s temperature is actively stabilized by a Peltier module and thus requires only air cooling. It has a fiber-delivered output connected to an SHG module, allowing for high flexibility in the arrangement of the TPEF-SLO system’s elements on a compact platform.

We also have shown that by minimizing the number of optical elements, we achieved a small chromatic dispersion of the TPEF-SLO (group delay dispersion of approximately 2800 fs^2^); thus, a compact dispersion precompensation device was enough to deliver ultrashort pulses, 76 fs, to the retina. Such a miniaturized TPEF-SLO easily fits on a 450 × 600 mm breadboard and could be placed on a movable platform, facilitating the ease of patient positioning during the imaging. Those advancements constitute an important step toward clinical translation of this method.

One of the significant challenges in this imaging modality is registering extremely weak fluorescence signals originating from the eye fundus while observing restrictive safety limitations to ensure noninvasive imaging. Furthermore, because of the numerical aperture (NA) difference between human and mouse eye imaging (0.1 and 0.4, respectively), a substantially lower signal was observed in humans, as expected (44). The intensity of TPEF is quadratically dependent on the average excitation power (Supplemental Information, *Fluorescence signal enhancement strategy*). However, the excitation power is restrained for safety reasons and limited to 0.3 mW. In such a case, other parameters of excitation light need to be carefully optimized. Our previous work has shown that shortening the pulse duration increases the fluorescence intensity and reduces the requisite laser power needed to visualize endogenous retinal fluorophores (19). Here, our laser provides ultrashort, 70-fs pulses with a clean temporal profile at its output, and almost no wings at the leading and trailing edges (Supplemental Figure 2), which would otherwise contribute to unwanted heating of the illuminated structure. Our TPEF-SLO is equipped with a dispersion precompensation unit, which allows us to deliver a short pulse with almost no change in the temporal profile directly to the retinal plane (as shown in Figure 1B) by canceling temporal broadening resulting from chromatic dispersion by optical components in the system and the eye.

Another modification that helped us in collecting informative images at low excitation power is the reduction of PRF. Reducing PRF at a given average excitation power translates to higher peak power, resulting in a higher fluorescence yield (18, 21). Our laser source allowed us to choose PRF within the 1 to 12 MHz range using an internal pulse picker. The 6 MHz PRF used here theoretically results in an over 13-fold increase in fluorescence signal with respect to 80 MHz PRF, which is the typical PRF of Ti:sapphire lasers.

The central wavelength of the laser source is a consequence of frequency-doubling the emission of the Er:fiber laser at 1560 nm. According to the absorption spectrum of retinal fluorophores, this wavelength fits the absorption spectra of A2E, flavin adenine dinucleotide (FAD), and melanin (16). However, shifting the excitation toward shorter wavelengths, e.g., 730 nm, enables visualizing retinyl esters (18, 19). Here, the central wavelength is induced by the emission spectrum of Er-doped media; however, shorter wavelengths can be generated by, e.g., dispersive wave generation in highly nonlinear fibers (45) preceding the SHG stage. An additional advantage of using a frequency-doubled laser is high spectral purity due to the selective SHG process. Thus, the laser light does not contain a broad spectral background that would potentially overlap with the transmission spectrum of emission filters in front of the PMT and contribute to background noise.

Lastly, a single image frame registered in the fluorescence channel did not contain enough photons to be informative. Averaging multiple frames was necessary to form images with sufficient SNR. Thus, it was necessary to correct for eye movement, which was done by simultaneous co-registration with the reflectance channel. Single frames in the reflectance channel had sufficient SNR to perform frame alignment and obtain transformation sets, which subsequently were applied to corresponding frames recorded in the fluorescence channel. After correction for eye motion and averaging approximately 100 frames, images were acquired with sufficient quality to visualize fundus features; thus, a larger number of frames was beneficial for image quality.

The TPEF images shown in Figure 2 provide an information-rich representation of the eye fundus in both analyzed ROIs. In each case, the recorded signal intensity was substantially higher than the noise floor of our system, containing dark counts and stray light. In front of the PMT, a set of emission filters was chosen to pass the fluorescence light only (within 400–700 nm spectral range) and exclude other possible light sources. Among those sources are an SHG (46, 47) and backscattering of excitation photons. The SHG can be excluded; given the spectrum of the laser, the SHG signal will have wavelengths shorter than 400 nm, which are outside the transmission spectrum of emission filters. The confocality for fluorescence photons was determined by the nonlinearity of the process. For backscattered photons, the PMT was nonconfocal because there was no mechanical pinhole. Furthermore, to prevent the excitation light backscattered on various layers of the eye, not limited to the retina and the RPE, from reaching the detector, we introduced 3 filters with a total OD of 20 at the spectral range of laser light. To determine that the backscattered light was entirely blocked, we performed the experiment with a CW laser of precisely the same average power in the pupil plane of the eye (0.3 mW) and similar central wavelength (central wavelength 825 nm; Supplemental Information and Supplemental Figure 6). No TPEF was expected with CW excitation at this power level. As shown in Figure 2, E and G, CW illumination resulted in a substantial (11.5-fold) decrease in signal intensity. No apparent fundus features were visible in the image. Still, the recorded signal intensity was 1.8 times higher than the noise floor of the measurement system. We attributed this signal to parasitic scattering of excitation photons by the eye fundus, though this signal was extremely weak (<0.008 pc/px). Interestingly, no scattering contribution was observed with other emission filters, where the signal recorded in the fluorescence channel with CW excitation was comparable to the noise floor. This experiment showed that the registered signal had a nonlinear nature, and we attributed the registered signal to TPEF.

Several significant differences between 1-photon and TPEF were noted (Figure 2). Due to the absorption spectra of retinal fluorophores, B-FAF is limited to imaging lipofuscin distribution (3). Excitation wavelengths shorter than 473 nm are not possible because of increased phototoxicity and absorption in the anterior segment of the eye. Both of those limitations can be bypassed by TPE in the NIR spectrum. Here, the excitation with a 780 nm central wavelength corresponds to 1-photon excitation with 390 nm light, which is expected to give access to other fluorophores, e.g., retinol and its derivatives, and NADH (16). Another difference that is apparent in our images is the lack of a dark spot in the fovea centralis. In B-FAF, the dark spot results from the absorption of blue excitation light by macular pigments (30). Here, we also bypass this absorption, which allows us to visualize the fundus structure at the macular region. This property confers the prospect for potential use of the method in monitoring photoreceptor dysfunction also in the macula. This feature of TPEF-SLO is similar to NIR-FAF (Figure 2G), which also omits the absorption of macular pigments; however, that method allows only for visualization of melanin distribution (31).

Avoiding the short-wavelength excitation of B-FAF has another important implication. Lipofuscin may be toxic to RPE, and short-wavelength illumination can increase its toxicity or increase the rate of its accumulation (48). In TPEF-SLO, this risk is mitigated. Another vital advantage of TPEF-SLO is its inherent confocality, obviating the need for a physical pinhole. This is especially important for fluorescence lifetime ophthalmoscopy, where lens autofluorescence is a critical problem (49). Finally, the use of NIR excitation causes significantly less patient discomfort than blue light in B-FAF, even at comparable values of average excitation power. The image pixel size should be comparable to the optical resolution (i.e., the size of the focused beam on the retina). Without adaptive optics, this size is large, approximately 20 μm, due to natural eye imperfections, and that is why our pixel size was set to approximately 21 μm. With the current safety standards, we could decrease this to smaller areas, but that would not help improve quality.

Retinol and retinyl esters are required for the formation of 11-*cis*-retinal, needed for visual pigment regeneration. However, defects in the function of proteins that maintain the retinoid cycle can lead to irregular quantities of these vitamin A–derived molecules and to blinding diseases such as Leber congenital amaurosis (50), or to aberrant production of retinal condensation products associated with retina degenerative Stargardt disease (14). We previously determined by HPLC that the content of retinyl esters in mouse eyes and primate eyes was very similar, on average approximately 4 pmol per mm^2^ of the retinal area. Furthermore, primate and mouse eyes had comparable amounts of A2E as measured by mass spectroscopy (51). As noted in this work, retinoid content in the human eye varies with the location within the eye. Retinoid-derived compounds differ in their fluorescence characteristics. Retinyl palmitate has a TPEF maximum at 515 nm; and A2E, representative of condensation products, at 650 nm (18). By using spectrally resolved TPEF-SLO we quantified TPEF spectral distribution from the fundus in humans and compared it with that measured in WT mice and mutant mice, models of retinopathies. We found that spectrally resolved TPEF from our human subjects was most similar to that measured in *Abca4–/–* *Rdh8–/–* mice, because it follows a similar pattern of intensity distribution across analyzed spectral ranges.

These results indicate that measurement of the differences in content and geographical location of retinoid-derived molecules could provide information about the aging retina and progress of the disease or the impact of therapy. Absorption spectra also differ among various types of retinal fluorophores (5). These differences raise the possibility that our future instrument could incorporate more than 1 fiber laser; for instance, one could be optimized for excitation of retinal condensation–type products and one for retinyl esters or retinol. However, this gain in versatility could come at the price of increasing the size and complexity of the instrument.

To determine the TPEF-SLO’s safety, we adopted the most restrictive limitation, namely, the MPE, expressed in J/cm^2^, calculated for a static beam and immobilized eye. We recalculated the resultant MPE to maximum permissible average radiant power (MPΦ_ave_), according to Delori et al. (27). Other ways of evaluating the potential hazard of SLO, which take into account the size of the illuminated retinal area, significantly increase the safety limit. For instance, we adopted 2 commonly known ways of simulating SLO, the pulsed line segment, and the pulsed minimal area and full-field approaches. The resultant MPE level was the lowest for the pulsed minimal area method, 14.1 mW. In the present study, no morphological or functional changes were observed after repeated exposures to TPEF-SLO laser light. We observed an anomaly in the RPE of subject 2 before and after exposure to TPEF-SLO laser light.

In conclusion, we present noninvasive optical imaging that can not only reveal important molecular mechanisms and uncover their anomalies before irreversible structural damage to retinal neurons has been detected, but can also help to accelerate the development of therapies against blinding diseases, which need to be arrested at the earliest stage. This demonstration is critical before advancing the TPE ophthalmic imaging to clinical assessment of early age- and disease-related changes in human retina and the impact of therapies.

## Methods

### Human subjects.

Two volunteers (45 and 44 years old) reported no visual problems and had no history of ocular diseases.

### Femtosecond fiber laser.

For this study, a custom femtosecond fiber laser with a tunable PRF was developed (21). The radiation at the 780 nm wavelength was obtained by frequency-doubling the Er-doped fiber laser output. The laser system consisted of 2 stages, an all-fiber femtosecond laser at 1560 nm and an SHG module, as schematically depicted in Supplemental Figure 2A. The first stage consisted of a low-power seed source, pulse picker, and 2 fiber amplifiers. The seed source was an Er-doped fiber laser mode locked via a semiconductor absorber mirror. It provided 1561 nm light, with an average power of 1.1 mW at 35.7 MHz PRF and 325-fs pulse duration. The 1561 nm light was amplified in a first Er-doped fiber amplifier up to approximately 10 mW and directed to a pulse-picker based on a fast acousto-optic modulator (52). The pulse-picker allowed reduction of the PRF from an initial 35.7 MHz to a value in a range of 1 to 12 MHz. Then, the signal with desired PRF was amplified in a second Er-doped fiber amplifier, which also introduced spectral broadening needed to produce ultrashort, 40-fs pulses at the output (45). Lastly, the pulses were compressed in a single-mode fiber (21). Thus, the first stage of the laser system generated pulses at the central wavelength of 1560 nm, a chosen PRF and 40-fs duration after compression (Supplemental Information and Supplemental Figure 2, B and C). More details on the fiber laser setup and a discussion on pulse shape evolution in the system can be found in our previous work (21). The output fiber was terminated with an FC/APC fiber connector for an easy connection with the second system stage, an SHG module. The beam from the fiber setup entered the SHG module through a collimating aspheric lens (C220TME-C, Thorlabs). Because of a quite broad bandwidth, the beam was then focused on a MgO:PPLN crystal (FSHNIR-ER, HC Photonics Corp.) by an achromatic lens with a focal length of 19 mm (AC127-019-C-ML, Thorlabs), which provided nearly constant focal length in the entire spectral bandwidth. The PPLN crystal had a 19.65 μm quasiphase matching period with poling lengths (*Lpoled*) from 0.1 to 1.0 mm (with a step of 0.1 mm) to choose from. After setup optimization, we chose *Lpoled* = 0.3 mm, providing the highest SHG efficiency, at a level of 23.3%. Then, the frequency-doubled signal was collimated by an aspheric lens (C560TME-B, Thorlabs) and directed at 2 filters. Filter 1 was a dichroic mirror reflecting the unconverted 1560 nm pump signal (DMSP1000, Thorlabs), and filter 2 was a bandpass filter with a cutoff at 700 nm (FELH0700, Thorlabs), which blocks higher harmonics below 700 nm. As a result, 70-fs pulses at 780 nm with a clean temporal shape were generated, as shown in Supplemental Figure 2, D and E. The laser provided a nearly constant pulse duration for all available PRFs (Supplemental Figure 2F).

### TPEF-SLO.

To precompensate for subsequent optical components’ dispersion in the setup, the femtosecond-laser’s output beam was guided through a prism pair compressor (AFS-SF10, Thorlabs, SF10 glass) (Supplemental Information and Supplemental Figure 1A). The 2 prisms were separated by 258 mm and placed at Brewster’s angle. An achromatic half-wave plate (AQWP10M-980, Thorlabs) was placed at the entrance to adjust the polarization. The double-pass configuration was accomplished by placing a slightly tilted mirror (M1) after the second prism. In this configuration, the compressor provided the estimated value of negative group-delay dispersion of –3270 fs^2^, and third-order dispersion of –10,985 fs^3^ (Supplemental Information and Supplemental Figure 1A), enough to precompensate for 2800 fs^2^ group-delay dispersion of the TPEF-SLO and internal dispersion of the laser source. These values were obtained by minimizing the width of the pulse autocorrelation function measured after the propagation through subsequent optical components in the setup, with an additional 23-mm glass cuvette filled with water, in the beam path. The water was used to mimic the dispersion of the human eye (53). This allowed optimization of the excitation pulse’s temporal properties in the retinal plane for the highest probability of 2-photon absorption. Another mirror (M2) was used to pick up the returning beam and direct it through the beam expander. The Galilean beam expander was composed of lenses L1 (LC1715-B-ML, Thorlabs, *f* = –50 mm) and L2 (LB1901-B-ML, Thorlabs, *f* = 75 mm) and provided a magnification of 1.5 and a 3 mm diameter beam (1/e^2^). According to diffraction theory, an increased beam size results in a smaller focal spot; however, the aberrations of the optical system of the eye counteract this relationship. Here, the beam size was chosen to minimize the spot size on the retina (40), which is directly related to TPEF intensity.

At the entrance to the TPEF-SLO, a shutter on a motorized flip mount (S, MFF101/M, Thorlabs) was placed and activated whenever the beam was not scanned. Next, a neutral density (ND) filter (NE08A, Thorlabs, OD = 0.8) was placed on another motorized flip mount to quickly adjust the input power. Next, the beam was directed to a pair of closed-coupled (i.e., 2 mirrors placed in perpendicular planes close to each other [8 mm separation]) galvanometer scanners (Saturn 5B, ScannerMAX) and a telescope composed of lenses L3 and L4 (AC254-075-B-ML, Thorlabs and AC254-075-AB-ML, respectively), thereby optically conjugating the scanners with the pupil plane of the examined eye. The position of lens L4 can be adjusted to correct for the variation in refraction properties of the imaged subject eye to maximize the fluorescence signal intensity. The maximum FOV of the system was 17.6°. Raster scanner images of the retina were acquired simultaneously in 2 channels, fluorescence and reflectance.

In the fluorescence channel, fluorescence emitted from the eye was separated from the excitation light using a dichroic mirror (DM, Multiphoton HC705LP, Semrock). An additional set of bandpass filters (BP, 2× 694/SP BrightLine HC, Semrock, and 1× FESH0700, Thorlabs) followed the DM to entirely remove the excitation and backscattered light and transmit the fluorescence light only (OD = 20, for spectral region of laser light, i.e., 760–840 nm, calculated based on measurement data of spectral transmission provided by the manufacturers). An exception is the experiment shown in Figure 3, where the third filter was exchanged with one of the following: Thorlabs FESH0600, MF620-52, FESH0550, or FBH520-40. A GaAsP photosensor of a PMT (H7422-40P, Hamamatsu, quantum efficiency of 45% at 600 nm) was placed in a plane conjugated to the pupil plane by a telescope made of lenses L4 and L5 (AC254-050-A-ML, Thorlabs). Photocurrent pulses from the PMT were fed to the photon-counting unit (PCU, C9744, Hamamatsu), which converted them to TTL pulses, counted by a data acquisition card (National Instruments PCIe-6321). The PCU also features a discriminator, which allows the rejection of dark counts.

In addition to serving as the fluorescence excitation source, the femtosecond laser was used to generate reflectance images, i.e., an SLO. The backscattered light was descanned and separated from the excitation light using a 50:50 nonpolarizing beam splitter (BS011, Thorlabs). The confocality was introduced by a focusing lens (L6, AC254-030-B-ML, Thorlabs) and multimode fiber (M42L01, Thorlabs, 50 μm core diameter, 0.22 NA), working as a detection pinhole. The light was then guided to the Si avalanche photodiode (APD, APD410A/M, Thorlabs), the output of which was registered using analog input in the data acquisition card.

Both channels, fluorescence and reflectance, were recorded simultaneously and further processed, as described in the *Image processing* section of the Supplemental Information and in Supplemental Figure 7. The images were acquired in a 256 × 256 pixel format with 20-μs pixel dwell time, resulting in a 0.76 frame/s rate. The pixel size was approximately 21 μm, which defined lateral resolution. Axial resolution was estimated using the FHWM of the squared illumination point spread function (54), which is equal to 130 μm FWHM in the eye. A customized LabVIEW software controlled the system. Open-access microscope control software served as a base for the development (55) of an improved scanning protocol and control of the device’s safety features.

### CW laser.

A semiconductor superluminescence diode (Superlum Broadlighter T870) was used as a CW excitation source. The average power of the light source was adjusted using a continuously variable ND filter to 0.3 mW in the pupil plane. The optical spectrum of the light source is shown in Supplemental Figure 6.

### Measurement procedure and examined subjects.

During the imaging session, a standard ophthalmic chinrest was used to stabilize the subject’s head. The chinrest was mounted on a translation stage, allowing adjustment of the eye’s position in lateral, vertical, and axial planes. The eye was fixed by a distant-spot fixation light seen by the fellow eye (630 nm light emitting diode, Supplemental Information and Supplemental Figure 2). The volunteer was covered with a black cloth, and imaging was performed in a dark room (<0.01 lux). The subject was not dark adapted before the imaging. The pupil was not dilated before the imaging session, except for images shown in Figure 4, where a visual field test was performed directly before the TPEF imaging. The ophthalmoscope-controlling software provided real-time visualization of a single frame each of the TPEF and reflectance images; however, a single TPEF image did not provide information sufficient for eye position adjustment. Therefore, the eye position was adjusted by looking at single-frame reflectance images, optimizing image sharpness and signal intensity. The position of lens L4 was adjusted to attain maximum fluorescence signal along the *z* axis of the eye. During this adjustment, the excitation power was kept low (0.05 mW) by inserting the ND filter in the motorized flip mount. After the eye position was found, the filter was removed, increasing the power up to 0.3 mW for image capture. A single imaging round lasted 40 seconds, and during this time, 30 frames were acquired. At least a 60-second break followed each round of data acquisition. This procedure was repeated 3 to 4 times (90–120 frames acquired) to obtain a sufficient number of frames for further processing.

### Clinical instruments.

To assess the safety of TPEF imaging, a set of clinical examinations was performed on the 2 subjects before and after the TPEF-SLO imaging. These tests included B-FAF and NIR-FAF by the Spectralis OCT Bluepeak module (Heidelberg Engineering), the 20° × 20° visual field test (nonmydriatic confocal microperimeter MAIA, Centervue), and OCT (Copernicus Revo, Optopol).

The scotopic visual field test was performed as detailed in the Supplemental Information.

### Animals.

Mice were housed in the animal facility in the University of California, Irvine Laboratory Animal Resources (ULAR) center. Mice were maintained on a normal mouse chow diet in a 12-hour light (~10 lux)/12-hour dark cyclic environment. Before imaging, mouse pupils were dilated with 1% tropicamide and mice were anesthetized with an intraperitoneal injection of ketamine (20 mg/mL) and xylazine (2 mg/mL) diluted with water at a dose of 5 μL/g body weight. WT mice, pigmented C57BL/6J mice, and BALB/cJ were obtained from The Jackson Laboratory. Albino and pigmented *Abca4−/−* *Rdh8−/−* double-knockout mice were generated as previously described (5, 56). *Rpe65−/−* mice were a gift from T. Michael Redmond (National Eye Institute, NIH, Bethesda, Maryland, USA). Male and female, 2- to 4-month-old mice were used in this study.

### TPEF mouse imaging.

TPE imaging and fluorescence lifetime data acquisition in mice were done as previously described (18). The imaging instrument was based on the Leica TCS SP8 Falcon architecture. A custom, tunable light source consisting of a Ti:sapphire laser (Vision S, Coherent) and a pulse selection system was used to generate 780 nm, 4–80 MHz IR light. A photodiode located after the pulse selection system provided synchronization signals for photon counting; thus, at each pixel, photon arrival time could be measured. A specially designed periscope objective was used to ensure large FOV and uniform excitation beam scanning on the retina and to maximize collection of fluorescence photons. During imaging, an anesthetized mouse was surrounded by a heated pad and placed on a mechanical stage with its eye covered with GenTeal gel and a thin 3.2 mm diameter, 0 diopter contact lens (Cantor and Nissel). During imaging, 1 to 3 mW of laser light was directed to the mouse eye. Fluorescence was measured as pixel mean gray value. Comparison of retinal fluorophores between mouse models and the arbitrary color scale for FLIM imaging were assigned based on the phasor approach (18).

### Data availability.

The authors declare that all data supporting the findings of this study are available within the paper and Supplemental Information.

### Study approval.

All procedures involving human subjects complied with the Declaration of Helsinki and were approved by the appropriate Ethics Committee of the Collegium Medicum, Nicolaus Copernicus University in Toruń (KB 313/2018). Signed informed consents were obtained before starting the procedures. All animal procedures were approved by the Animal Care Committee at the University of California, Irvine, protocol number AUP-21-096, and conformed to the recommendations of both the American Veterinary Medical Association Panel on Euthanasia and the Association for Research in Vision and Ophthalmology.

### Statistics.

Data from at least 3 independent experiments are presented as mean ± SD. Additional details can be found in the figure legends.

## Author contributions

JB, GP, KP, and MW designed the study. JB, GP, K Komar, KP, PK, and MW designed experiments. JB, JM, PK, MJM, and BLS performed experiments on human subjects. GP performed experiments on mice. JB, GP, ST, PK, K Komar, MJM, BLS, KP, and MW analyzed the data. DS, AH, AG, ZŁ, and GS designed, built, and characterized the femtosecond fiber laser. GS, K Karnowski, KP, and MW supervised the project. JB, GP, K Komar, KP, and MW wrote the manuscript. JB and GP are designated as co–first authors; JB is listed as first author because of his critical involvement with TPEF-SLO tests in human subjects. All authors contributed to writing and editing the manuscript.

## Supplementary Material

Supplemental data

ICMJE disclosure forms

## Version 1 11/30/2021

In-Press Preview

## Version 2 01/18/2022

Electronic publication
