# Supplementary material for: In vivo imaging of the human eye using a 2-photon-excited fluorescence scanning laser ophthalmoscope
Source: J Clin Invest. 2022 Jan 18;132(2):e154218. doi: 10.1172/JCI154218 (PMC8759795; doi:10.1172/JCI154218)
Supplement: Supplemental data [file jci-132-154218-s005.pdf]

SUPPLEMENTARY INFORMATION

In vivo Imaging of the Human Eye Using a Two-Photon Excited Fluorescence Scanning Laser Ophthalmoscope

Jakub Bogusławski[1,2,3,#], Grazyna Palczewska[1,2,4,5,#], Slawomir Tomczewski[1,2], Jadwiga Milkiewicz[1,2], Piotr Kasprzycki[1,2], Dorota Stachowiak[3], Katarzyna Komar[1,2,6], Marcin J. Marzejon[1,2,7], Bartosz L. Sikorski[8,9], Arkadiusz Hudzikowski[3], Aleksander Głuszek[3], Zbigniew Łaszczych[3], Karol Karnowski[1,2], Grzegorz Soboń[3], Krzysztof Palczewski[5,10]*, Maciej Wojtkowski[1,2,6]*

[#] co-first authors

[1]International Center for Translational Eye Research, Polish Academy of Sciences, Warsaw, Poland

[2]Department of Physical Chemistry of Biological Systems, Institute of Physical Chemistry, Polish Academy of Sciences, Warsaw, Poland

[3]Laser & Fiber Electronics Group, Faculty of Electronics, Wrocław University of Science and Technology, Wroclaw, Poland

[4]Polgenix, Inc., Department of Medical Devices, Cleveland, OH 44106, USA

[5]Gavin Herbert Eye Institute, Department of Ophthalmology, University of California, Irvine, CA 92697, USA

[6]Faculty of Physics, Astronomy and Informatics, Nicolaus Copernicus University, Torun, Poland

[7]Department of Metrology and Optoelectronics, Faculty of Electronics, Telecommunications and Informatics, Gdansk University of Technology, Gdansk, Poland

[8]Department of Ophthalmology, Nicolaus Copernicus University, Bydgoszcz, Poland

[9]Oculomedica Eye Research & Development Center, Bydgoszcz, Poland

[10]Department of Physiology & Biophysics, School of Medicine, and Chemistry, Molecular Biology and Biochemistry, University of California, Irvine, CA 92697, USA

[*]To whom correspondence should be addressed

CONTENT

Supplementary Figure S1. Experimental setup of TPEF-SLO.

Supplementary Figure S2. Frequency-doubled femtosecond Er:fiber laser.

Supplementary Figure S3. Axial resolution of TPEF imaging.

Supplementary Figure S4. Optical scheme of one- and two-photon perimeter.

Supplementary Figure S5. Scotopic visual field test utilizing one- and two-photon perimetry.

Supplementary Figure S6. Comparison of optical spectra of SNG and CW lasers.

Supplementary Figure S7. Alignment of consecutive frames acquired with TPEF-SLO.

Supplementary Figure S8. Reducing the pulse repetition rate allows to increase the TPEF signal at maintained average excitation power.

Supplementary Table S1. TPEF-SLO safety limits expressed as MPΦ.

Safety considerations

Scotopic visual field test

Fluorescence signal enhancement strategy

References

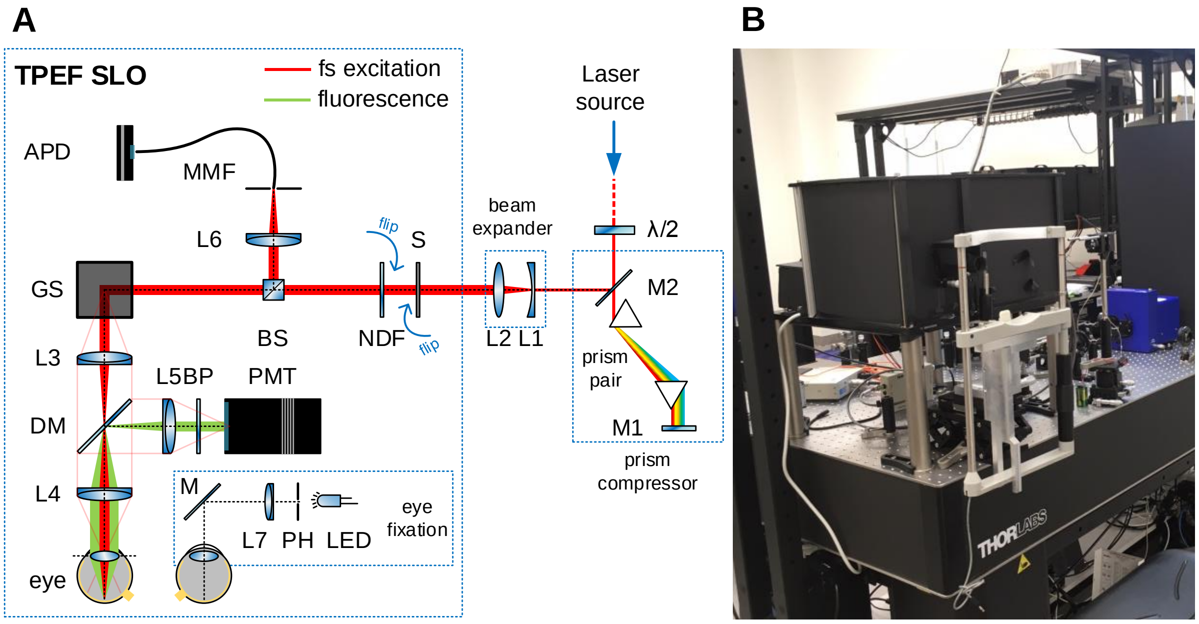

Supplementary Figure S1. Experimental setup of TPEF-SLO. (A) Scheme of the setup. λ/2 – half-wave plate, M - mirrors, L - lenses, S - mechanical shutter, NDF - neutral density filter, BS – beam splitter, GS - galvanometer-based xy scanners, DM - dichroic mirror, BP - set of bandpass filters, PMT - photomultiplier tube, MMF - multimode fiber, APD - avalanche photodiode, LED - light-emitting diode, PH - pinhole. (B) Photograph of the laboratory setup.

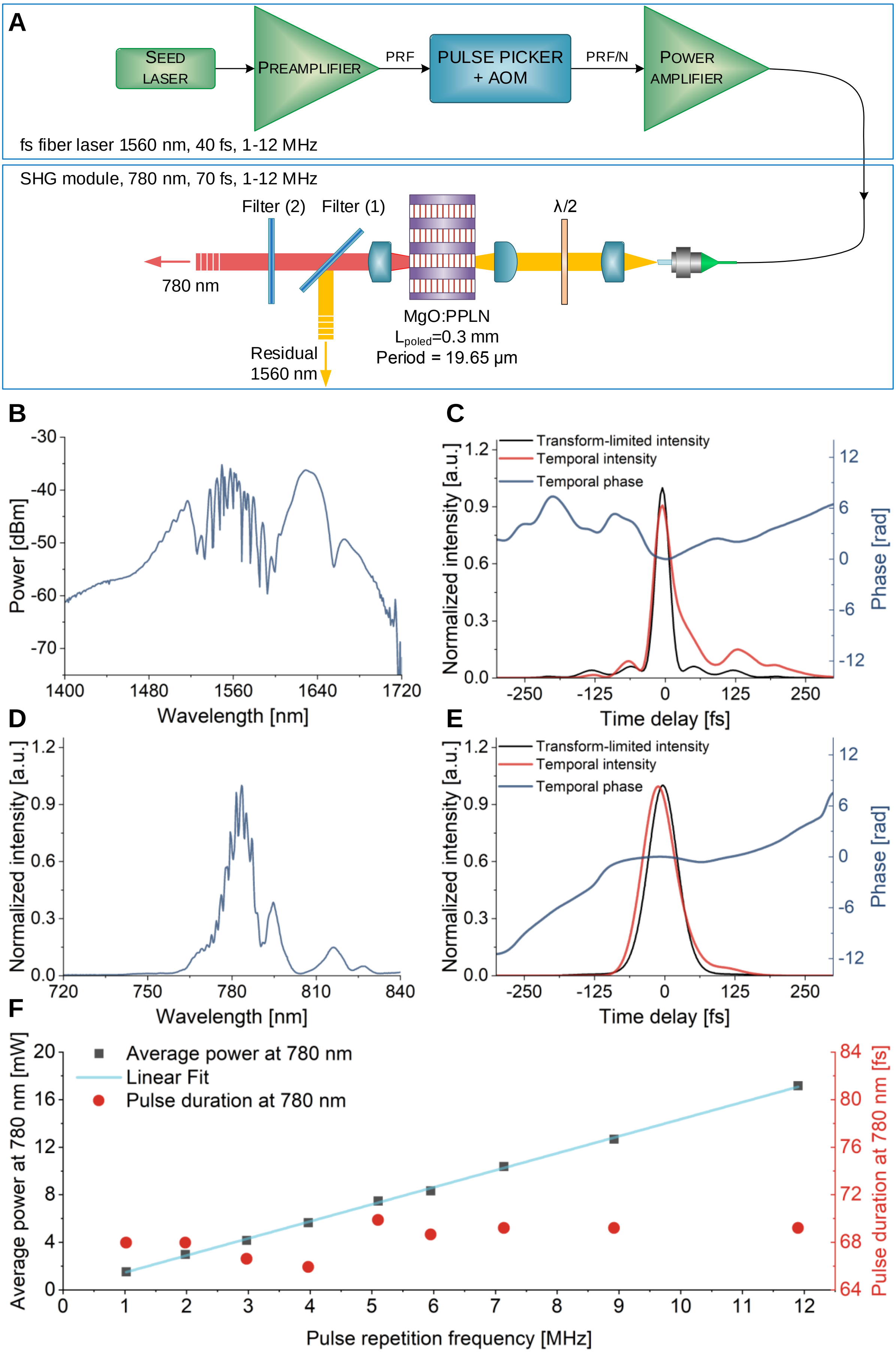
A
SEED LASER
PREAMPLIFIER
PRF
PULSE PICKER + AOM
PRF/N
POWER AMPLIFIER
fs fiber laser 1560 nm, 40 fs, 1-12 MHz
SHG module, 780 nm, 70 fs, 1-12 MHz
Filter (2)
Filter (1)
λ/2
780 nm
Residual 1560 nm
MgO:PPLN
Lpoled=0.3 mm
Period = 19.65 µm
B
Power [dBm]
Wavelength [nm]
C
Transform-limited intensity
Temporal intensity
Temporal phase
Normalized intensity [a.u.]
Phase [rad]
Time delay [fs]
D
Normalized intensity [a.u.]
Wavelength [nm]
E
Transform-limited intensity
Temporal intensity
Temporal phase
Normalized intensity [a.u.]
Phase [rad]
Time delay [fs]
F
Average power at 780 nm
Linear Fit
Pulse duration at 780 nm
Average power at 780 nm [mW]
Pulse duration at 780 nm [fs]
Pulse repetition frequency [MHz]

Supplementary Figure S2. Frequency-doubled femtosecond Er:fiber laser. (A) Scheme of frequency-doubled femtosecond Er:fiber laser with tunable pulse repetition frequency (PRF); AOM – acousto-optic modulator, λ/2 – half-waveplate, PPLN – periodically poled lithium niobite, L_{poled} – poling period length, SHG – second harmonic generation. (B-C) Optical spectrum and temporal intensity of the pulse (solid red line) together with the calculated transform-limited intensity (solid black line) and temporal phase (solid blue line); all measured at 5.94 MHz PRF. (D-E) Optical spectrum and temporal intensity of the pulse (solid red line) together with the calculated transform-limited intensity (solid black line) and temporal phase (solid blue line); all measured after the SHG module at 5.94 MHz PRF. Temporal intensity of the pulse was retrieved using the frequency-resolved optical gating (FROG) method (FROGScan Ultra2, Mesa Photonics) (1) (F) Average output power and pulse duration after the SHG as a function of PRF.

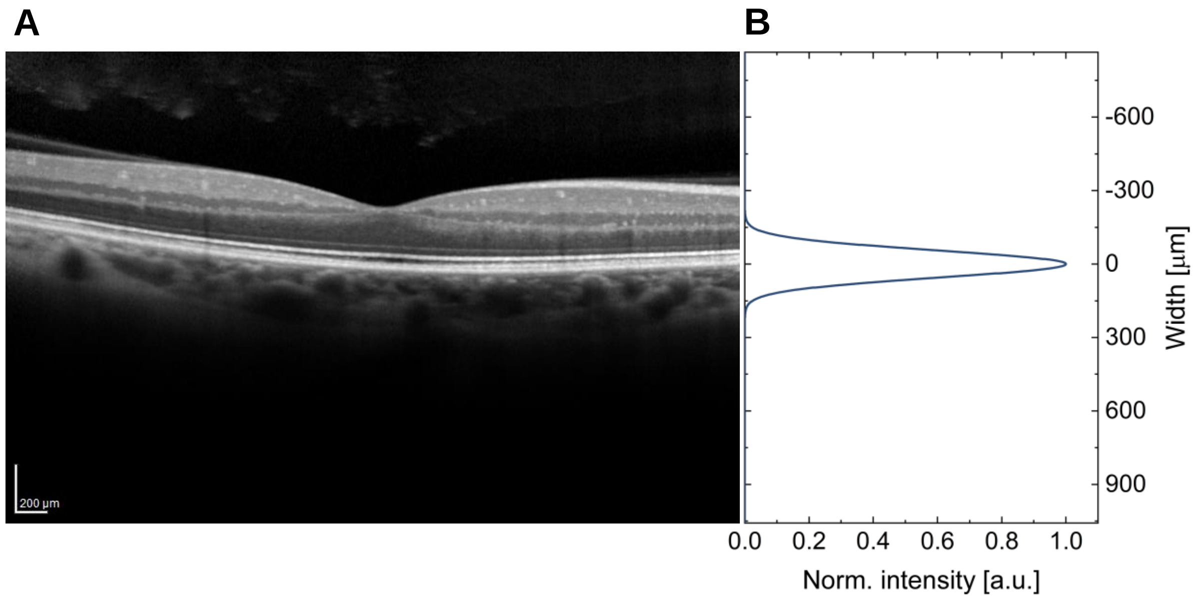

Supplementary Figure S3. Axial resolution of TPEF imaging. Shown is the juxtaposition of a cross-section of the retina obtained in a Fourier-domain (FD) OCT with $IPSF^2_z$ estimated by Gaussian function centered at the RPE layer. (A) FD-OCT retinal image. (B) Axial illumination point spread function squared ($IPSF^2_z$) estimated by a Gaussian function with peak coinciding with RPE layer. To estimate which retinal layers may contribute to the TPEF signal measured, we calculated the width of $IPSF^2_z$ (2). Assuming a 0.1 numerical aperture of the eye for a 3 mm input beam and vitreous' refractive index of 1.336 (3), we calculated the full width at half maximum (FWHM) of $IPSF^2_z$ to be 130 µm. Focal depth covers both the photoreceptor layer (PR) and RPE, also reaching the neighboring layers: outer nuclear layer, plexiform layer, and Bruch's membrane. However, the TPEF signal is expected to originate from the fluorophore-containing layers, *i.e.,* PR and RPE.

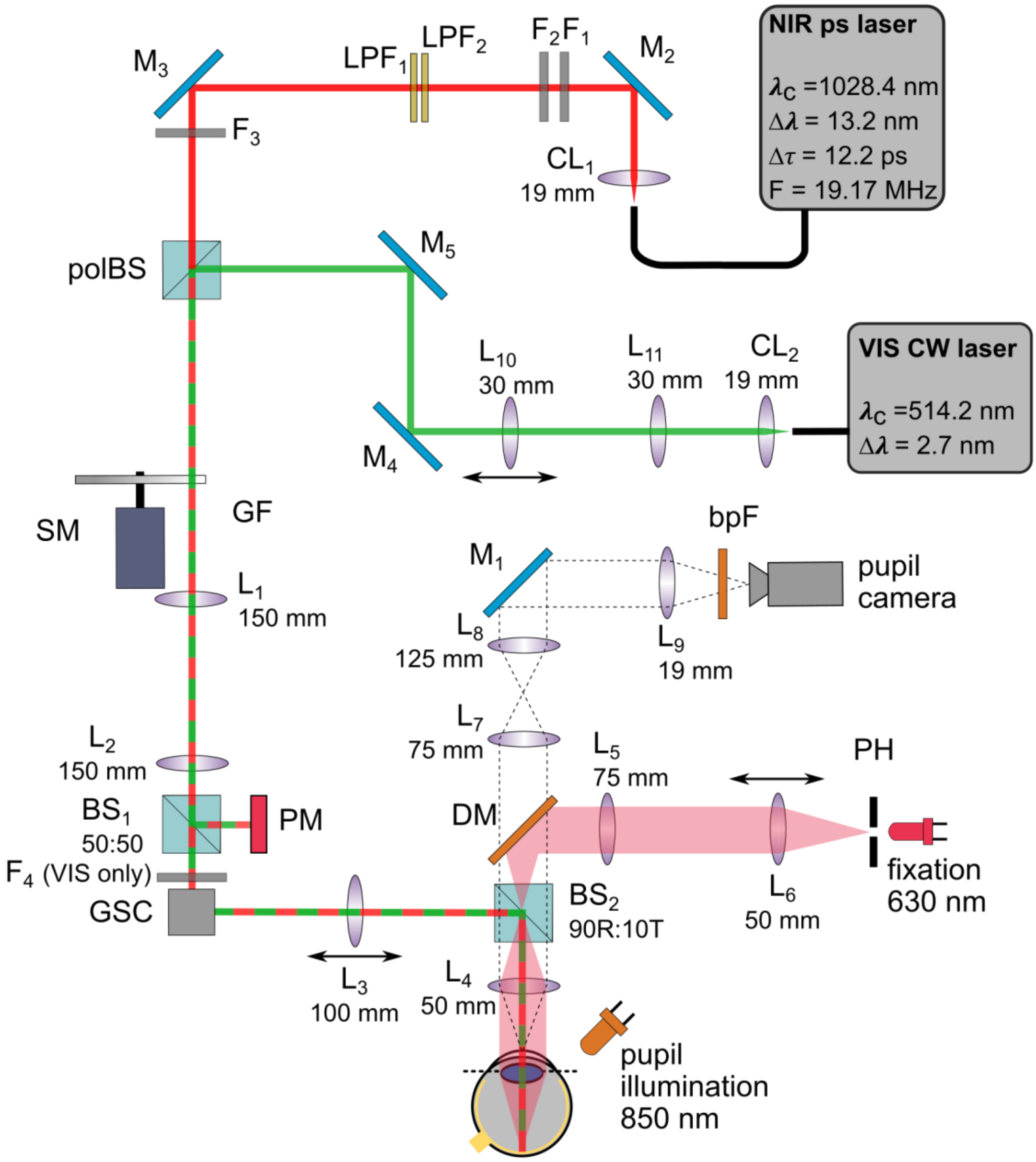

Supplementary Figure S4. Optical scheme of one- and two-photon perimeter. Symbols: BS_i – beam-splitter, bpF – bandpass filter, CL_i – collimating lens, DM – dichroic mirror, F_i – neutral density filter, GF – neutral density gradient filter, GS – galvanometric scanners, L_i – lens, focal lengths are indicated in the scheme, LPF_i – long-pass filters 850 nm cut-off, M_i – mirror, PM – power meter, PH – pinhole, polBS – polarization beamsplitter, SM – stepper motor, VIS – visible.

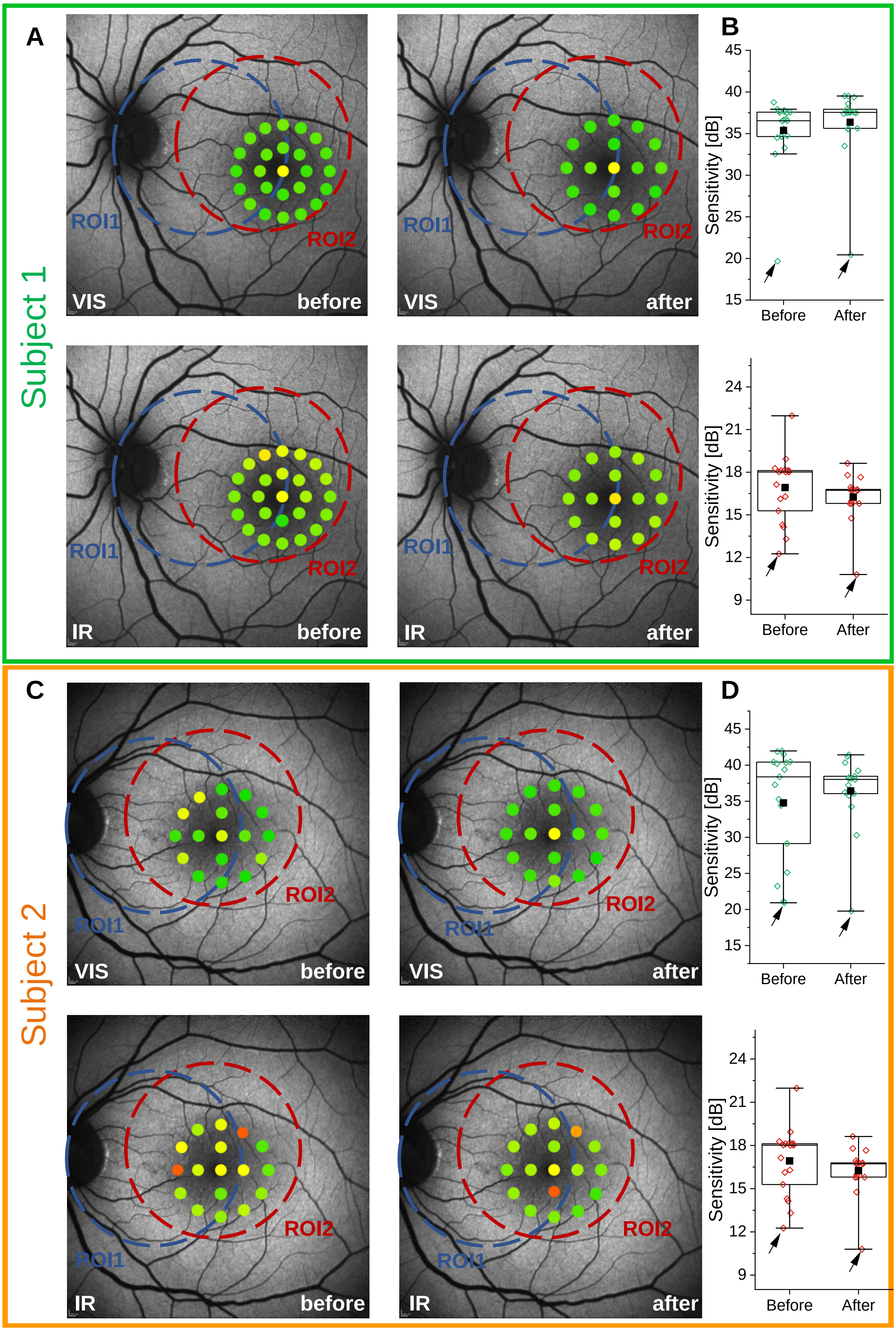
A
B
C
D
Subject 1
Subject 2
ROI1
ROI2
VIS
IR
before
after
Sensitivity [dB]
Before
After

Supplementary Figure S5. Scotopic visual field test utilizing one- and two-photon perimetry. The visual field was tested before and after TPEF imaging, using visible (VIS) and infrared (IR) stimuli for two subjects. ROI1 and ROI2 correspond to retinal regions outlined in TPEF-SLO images shown in Figure 2. (**A**) Sensitivity maps for subject 1; tested before and after recording 200 frames in TPEF-SLO; number of tested retinal locations slightly differ before and after, because the number of tested points had to be reduced due to subject's fatigue. Sensitivity maps are indicated over B-FAF images, shown in Figure 2I. (**B**) Visual sensitivities measured with VIS (upper row) and IR stimuli (bottom row) before and after imaging with TPEF-SLO for subject 1 (n=21, before, n=17, after; 1-way ANOVA, P>0.05 in all). (**C**) Sensitivity maps for subject 2; tested before and after recording 400 frames in TPEF-SLO. Sensitivity maps are plotted over B-FAF images, previously shown in Figure 4B. (**D**) Visual sensitivities measured with VIS (upper row) and IR stimuli (bottom row) before and after imaging in TPEF-SLO for subject 2. (n=17, before and after; 1-way ANOVA, P>0.05 in all). Quantitative data are represented as a box-and-whisker plots, with bounds from 25th to 75th percentile, median line, mean value (black square), and whiskers ranging from 5 to 95 percentile of sensitivity values. Black arrows indicate sensitivity measured at the fovea center, characteristically lower in scotopic conditions.

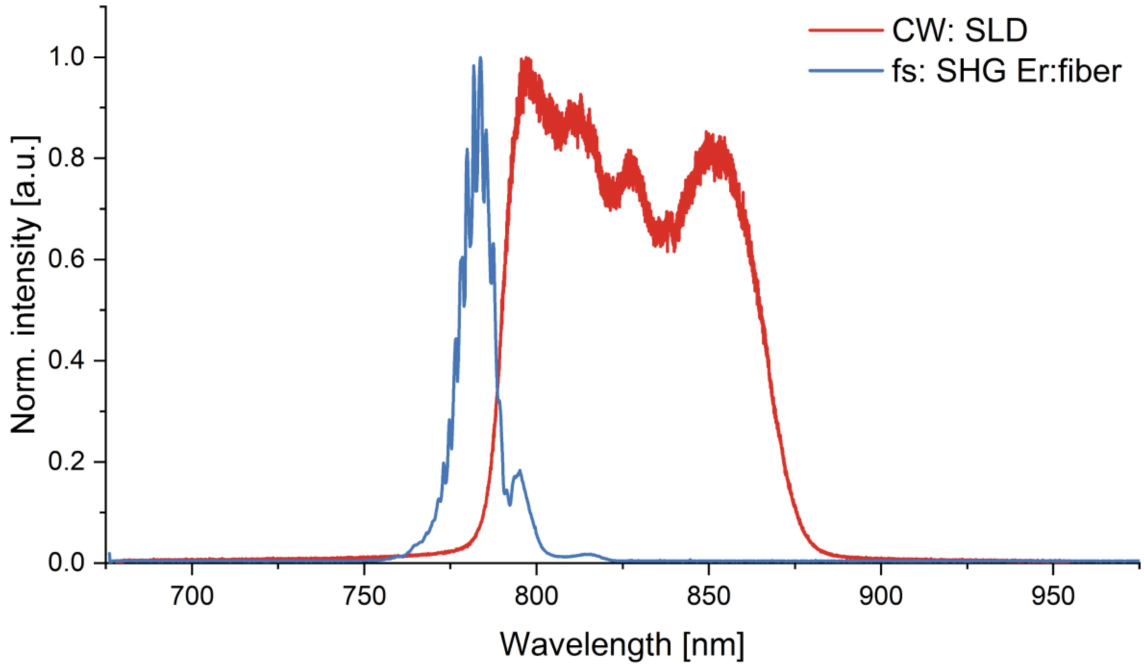

Supplementary Figure S6. Comparison of optical spectra of SHG and CW lasers. SHG Er:fiber, femtosecond laser; CW laser, superluminescence laser diode.

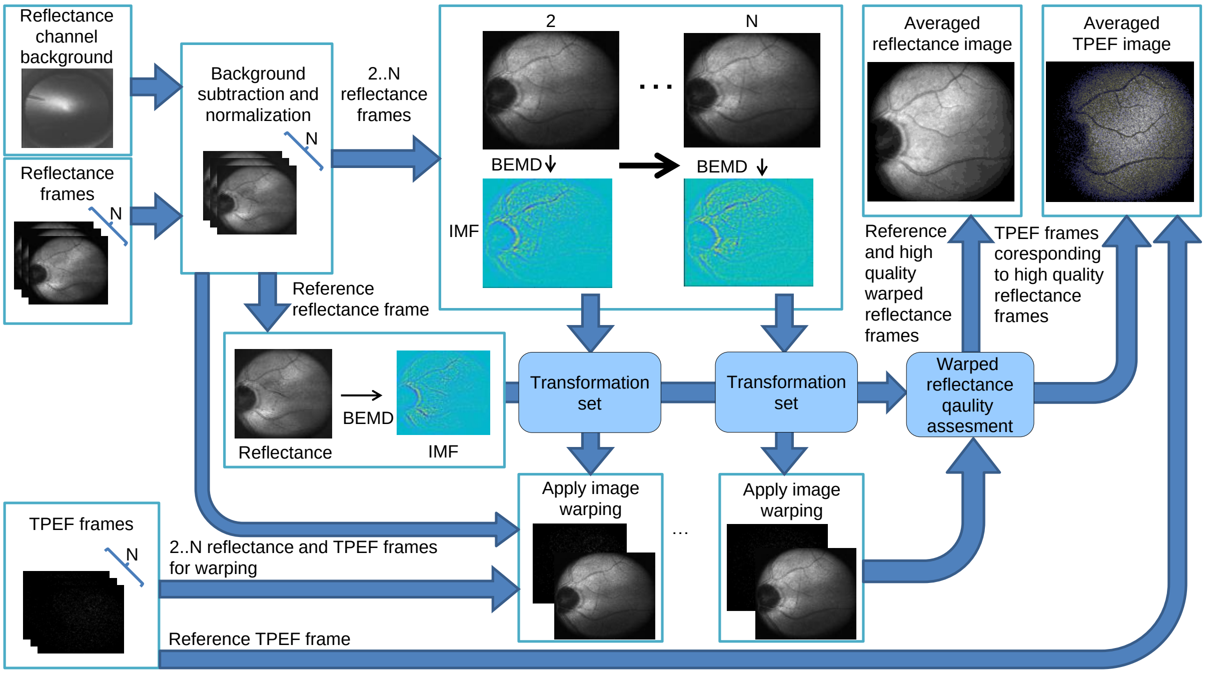

Supplementary Figure S7. Alignment of consecutive frames acquired with TPEF-SLO. The SLO frames are used for the calculation of displacement fields. Next, SLO and TPEF images are warped. The output images are calculated as averages of warped SLO and TPEF frames. IMF - Intrinsic Mode Functions; BEMD – bi-dimensional Empirical Mode Decomposition.

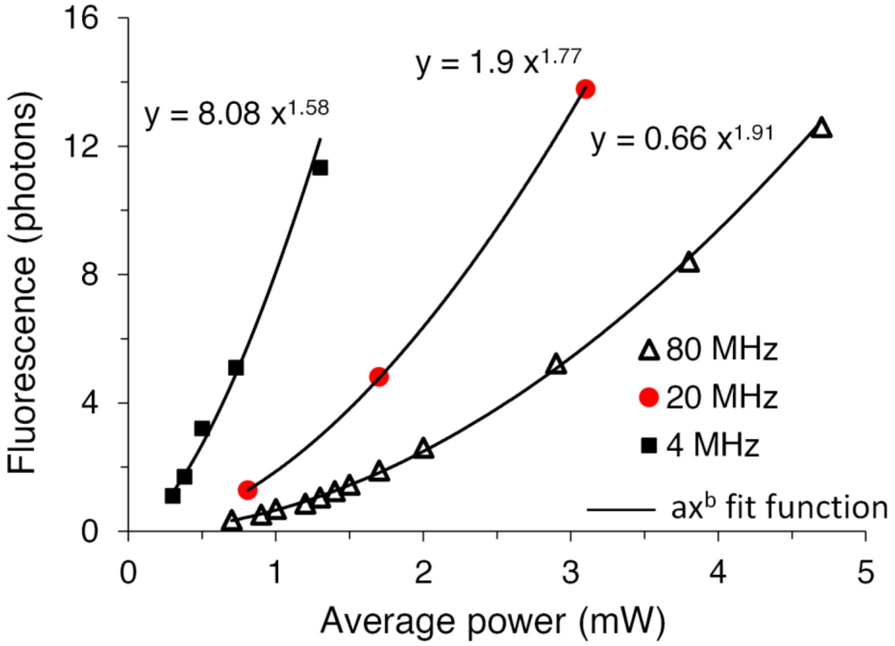

Supplementary Figure S8. Reducing the pulse repetition rate allows to increase the TPEF signal at maintained average excitation power. Average fluorescence per image from *Abca4*$^{-/-}$*Rdh8*$^{-/-}$ mouse eyes at 80, 20, and 4 MHz PRFs.

Supplementary Table S1. TPEF-SLO safety limits expressed as MPΦ. TPEF-SLO safety limits expressed as Maximum Permissible average Radiant Power (MPΦ) determined for the retinal exposures to 780 nm light in this study and for the exemplary conditions.

Retinal exposures (RE) for beam of average power 0.3 mW (exposure duration, number of frames)		TPEF-SLO safety limits [mW]			Static beam (4) [mW]
		PLS (1)	PMA (2)	FF (3)	
this study 17.6°x17.6°	0.044 J/cm^2 (40 s, 30 frames)	21.1	14.1	250.5	0.398
	0.176 J/cm^2 (160 s, 120 frames)	21.1	14.1	177.1	0.281
	1.76 J/cm^2 (1600 s, 1200 frames)	21.1	14.1	99.6	0.158
exemplary conditions 1.5°x1.5°	6 J/cm^2 (40 s)	21.1	7.6	6.9	0.398

Safety considerations

To determine the TPEF-SLO's safety regime, we tested several approaches: (1) *the pulsed line segment (PLS) simulation*, described in (4); (2) similar but more restrictive *pulsed minimal area (PMA) simulation;* and (3) *the full-field (FF) simulation* (4). For comparison we also provide the limits calculated for (4), the *static beam* (5), as well as SLO safety limits calculated for the smaller retinal area (1.5°x1.5°), close to the isoplanatic patch of the eye (6) and useful for comparison with previously published data. The numerical values of calculated limits are provided in **Supplementary Table 1**.

The laser used for TPE has the central wavelength of λ = 780 nm, the PRF of F_{laser} = 5.94 MHz, and pulse duration τ_{pulse} = 76 fs. The imaged retinal area was a square of α_F = 17.6° per side (307 mrad). Our scanning pattern was a raster of R = 256 lines, without blanked return, the frequency of fast scanner was F_{fast} = 97.67 Hz (t_{line} = 5.12 ms), and the time required for acquisition of one frame was t_{frame} = 1.31 s (F_{frame} = 0.76 Hz). The single measurement lasted T_{single} = 40 s and allowed to gather 30 frames. To obtain 100-120 frames for the high quality TPEF image, 4 single measurements were required, separated by at least 60 s of blanked beam for subject rest; therefore, total exposure time for measurement was T_{day} = 160 s. Taking into account that some images presented in this paper required several sessions on different days (separation time between these days ranged from 1 day to several days), we also calculated total exposure of the eye assuming time T_{total} = 1,600 s.

To compare with previously published safety assessments of near-IR laser exposures for scanning laser imaging (7-9), the retinal exposures were calculated as follows (4):

$$RE = \frac{P \cdot T_{exp}}{A_{retina}}$$

where P is average excitation power and T_{exp} is exposure time. A_{retina} is the area of exposed retina defined as:

$$A_{retina} \approx \frac{\pi}{4}(f_e \alpha)^2$$

where f_e is the focal length of the human eye (17 mm, Gullstrand's model eye) and α is the full angle of exposed area in radians (in our case 346 mrad - the diameter of the circular area, equivalent to the square 307 mrad x 307 mrad). Under these assumptions, the retinal exposure with 0.3 mW of excitation power and exposure time T_{single} = 40 s is RE_{single} = 0.044 J/cm^2 for a single measurement; while RE_{day} = 0.176 J/cm^2 for T_{day} = 160 s, and finally RE_{total} = 1.76 J/cm^2 for T_{total} = 1,600 s of repeated exposures of the human eye. All of these exposures were much lower than exposures causing a reversible long-term decrease of NIR-FAF observed in previous studies (*e.g.* 15 J/cm^2 in (8) and 20.4 J/cm^2 in (9)).

(1) The pulsed line segment simulation is based on the value of t_{min} = 5 μs provided by the ANSI standard: the time during which heat transfer from the exposed site is sufficiently small to assume that all energy delivered in this period of time was supplied in the form of one pulse of duration t_{min} (4). During this period of time, the laser beam scanning the retina forms the line of angular length α_S:

$$\alpha_S = \frac{t_{min}}{t_{line}}\alpha_F = 0.3\, mrad$$

Another value provided by the ANSI standard is the minimal retinal area α_{min} = 1.5 mrad. This size is larger than a transverse point-spread function of the eye, but it is assumed that small-angle forward scattering effectively enlarges the size of the minimal possible thermal lesion to the α_{min} (4). Therefore the laser beam returning in the next line (or in the *m* next lines) may effectively visit the same retinal spot of size α_{min} *m* times. Furthermore, considering that in safety calculations, we always round-up to the worst case, *m* is equal to:

$$m = \frac{\alpha_{min}}{\alpha_F}R = 1.25 \approx 2$$

Thus each consecutive frame is equivalent to exposure of retinal area for m = 2 pulses of duration t_{min} = 5 μs, separated by time t_{line} = 5.12 ms, which gives the $t_{group} = mt_{line}$ = 10.24 ms. Each single measurement lasted T_{single} = 40 s and consisted of n = 31 frames (rounding up):

$$n = F_{frame}T_{single} \approx 31$$

for longer exposure times (T_{day} and T_{total}) the number *n* will be larger, accordingly equal to 123 and 1221.

The raster scanning is equivalent in this approach to the multiple pulse exposure described in paragraph 8.2.3 of the ANSI standard and the relevant 3 Rules provided there should be evaluated to the group of *m*-pulses and to the train of *n*-groups of pulses. The most restrictive limit should be applied. Considering the small size of the segment ($\leq\alpha_{min}$), the Rule 3 will be always equal to Rule 1 in our case (paragraph 8.2.3 and Table 6c). Rule 1 checks the single pulse limit:

$$MPE_{single,group}\left[\frac{\mathrm{J}}{\mathrm{cm}^2}\right] = 1.8 \cdot C_A \cdot C_E \cdot t_{min}^{0.75} \cdot 10^{-3} \cong 275\frac{\mathrm{nJ}}{\mathrm{cm}^2}$$

The C_A correction factor for the 780 nm wavelength is equal to 1.4454 (Table 6a) and C_E correction factor is equal to 1 due to the size of the segment $\leq\alpha_{min}$ (Table 6b).

Rule 2 verifies the average power limit:

$$MPE_{average,group}\left[\frac{\mathrm{J}}{\mathrm{cm}^2}\right] = \frac{1.8 \cdot C_A \cdot C_E \cdot t_{group}^{0.75} \cdot 10^{-3}}{m} \cong 41.9\frac{\mathrm{mJ}}{\mathrm{cm}^2}$$

Similarly, for the train of n groups the appropriate single-pulse limit and average power limit can be calculated as follow:

$$MPE_{single,train}\left[\frac{\text{J}}{\text{cm}^2}\right]=\frac{1.8\cdot C_A\cdot C_E\cdot t_{group}^{0.75}\cdot 10^{-3}}{m}= MPE_{average,group}\cong 41.9\frac{\text{mJ}}{\text{cm}^2}$$

and:

$$MPE_{average,train}\left[\frac{\text{J}}{\text{cm}^2}\right]=\frac{1.8\cdot C_A\cdot C_E\cdot T_{single}^{0.75}\cdot 10^{-3}}{n\cdot m}\cong 667.5\frac{\text{mJ}}{\text{cm}^2}$$

The most restrictive is Rule 1 for the $MPE_{single,\ group}$. For longer exposures T_{day} and T_{total}, the $MPE_{average,train}$ decreases, however still $MPE_{single,group}$ remains the most restrictive limit as being three orders of magnitude lower.

The MPE limit in [J/cm^2] determined by pulsed line segment simulation can be recalculated to the MPΦ in [W] or PLS (1) in **Supplementary Table 1** [1]:

$$MP\Phi_{ave}[W]=\frac{MPE\cdot A_p}{t_{min}}\cong 21.1\text{ mW}$$

where A_p is the pupil area (A_p = 0.385 cm^2).

(2) *The pulsed minimal area simulation* is similar to that presented above, except that the size of retinal area exposed to multiple returns of scanning laser beam is confined to α_{min} in both dimensions. This area is visited *m*-times by the scanning laser beam during one frame and *n*-times during the whole exposure time. The exposure of each minimal area to the scanning laser beam can then be treated as the exposure to a train of pulses of duration t_0:

$$t_0=\frac{\alpha_{min}}{\alpha_F}\cdot t_{line}\cong\ 25\ \mu\text{s}$$

The *m* and *n* values are the same as in the pulsed line segment simulation described above. The scanning raster of our SLO was equivalent to exposure of the eye for *n* = 31 groups of *m* = 2 pulses of duration t_0 = 25 μs; the pulses in the group were separated by t_{line} = 5.12 ms (the group duration was again t_{group} = 10.24 ms), and the groups of pulses were separated by T_{frame} = 1.31 s. For these conditions the ANSI rules for multiple pulse exposures were evaluated and again the most restrictive was Rule 1 for the group of *m* pulses:

$$MPE_{single,group}\left[\frac{\text{J}}{\text{cm}^2}\right]=1.8\cdot C_A\cdot C_E\cdot t_0^{0.75}\cdot 10^{-3}\cong 919\frac{\text{nJ}}{\text{cm}^2}$$

After converting this limit to a more practical MPΦ one obtains:

$$MP\Phi_{ave}[W]=\frac{MPE\cdot A_p}{t_0}\cong 14.1\ m\text{W}$$

(3) *The full-field simulation* assumes that the whole retinal area illuminated by a scanning beam within one frame is treated as a homogeneously illuminated field (4). Therefore the exposure for extended sources applies, according to Table 5f [2]:

$$MPE\left[\frac{\mathrm{J}}{\mathrm{cm}^2}\right] = 1.8 \cdot C_A \cdot C_E{}^{*} \cdot T_{exp}^{0.75} \cdot 10^{-3} \cong= 26.03\frac{\mathrm{J}}{\mathrm{cm}^2}$$

with the C_E^* correction factor for the squared source of 307mrad x 307mrad size equal to 629 (Table 6b) [2]. This limit could be again recalculated to the MPΦ, or FF (3) in **Supplementary Table 1**:

$$MP\Phi_{ave}[W] = \frac{MPE \cdot A_p}{T_{exp}} \cong 250.5\ \mathrm{mW}$$

The MPΦ values obtained for longer exposure times (T_{day} and T_{total}) are provided in **Supplementary Table 1**. For pulsed light illumination requiring the beam to be focused to a small size, this method of analysis is not the best way to assess the safety of using an optical device in a clinical setting and therefore should not be considered further.

(4) *The static beam case*. At the end we adopted the most restrictive limitation: the Maximum Permissible Exposure (MPE) calculated for the hypothetical case of a static beam and an immobilized eye for the time period T_{exp} = 40 s, sufficient to gather 30 image frames. A single imaging session consisted of several such trials lasting 30-40 s, separated by breaks for resting of the subject, which lasted at least a minute each. Due to the high repetition frequency (F_{rep} = 5.94 MHz) according to Table 1 of ANSI, we have to evaluate only Rule 2 (Average Power MPE). The cumulative MPE for 780 nm stationary beam of 3 mm diameter (smaller than pupils of the examined subjects) was then calculated from Table 5c [2] as:

$$MPE\left[\frac{\mathrm{J}}{\mathrm{cm}^2}\right] = 1.8 \cdot C_A \cdot T_{exp}^{0.75} \cdot 10^{-3} \cong 41.38 \cdot 10^{-3}\frac{\mathrm{J}}{\mathrm{cm}^2}$$

The resulting MPE limit is then equal to:

$$MP\Phi_{ave}[W] = \frac{MPE \cdot A_p}{T_{single}} \cong 0.398\ \mathrm{mW}$$

Using this model, we also calculated the MPE for the very unlikely case of scanner failure. Our TPEF-SLO system is shutdown in less than 5 s in the case of scanner failure, which corresponds to MPE of 0.67 mW.

Figure 1D shows the retinal MPE for a static beam (MPE_r, red solid line) as a function of exposure time. MPE_r was obtained by dividing the $MPE \cdot A_p$ by the area of illuminated retina in the presence of eye movement. A circular area with a diameter of approx. 190 μm (visual

angle of 11 mrad) was used, as recommended by the Standard for long exposure durations (4).

We also calculated the single-pulse MPE limit (MPE_{SP}) per ANSI for 100 fs pulses. For 100 fs pulses, Table 5c provides the single pulse formula, MPE_{SP} = 1.0 x 10^{-7} J/cm^2 and paragraph 8.3.5 halves this value for the immobilized eye and number of pulses greater than 600. After multiplying by the pupil area equal to 0.385 cm^2, MPE_{SPA} = 1.925·10^{-8} J. This MPE, in terms of average irradiance (MPE:E) is equal to:

$$MPE{:}E = MPE_{SP} \text{ x } PRF = 0.5 \text{ x } 10^{-7} J/cm^2 \text{ x } 5.94 \text{ x } 10^6 \ s^{-1} = 0.297 \ W/cm^2$$

After multiplication by the PRF and the pupil area, the MPE_{SP} can be linked to power measured at the cornea plane, P_{maxSP}= 0.5 x 10^{-7} J/cm^2 x 5.94 x 10^6 s^{-1} x 0.385 cm^2 = 0.114 W or 114 mW. Thus, single- pulse MPE is not our limiting value.

Fluorescence signal enhancement strategy

In two-photon fluorescence excitation with pulsing light, fluorescence (*F*) is proportional to the square of average power (P_{avr}), as shown by the following equation:

$$F \propto K \cdot \frac{g_p}{PRF \cdot \tau} \cdot P_{avr}^2$$

, where *K* is the constant dependent on type and concentration of fluorophore, wavelength and the collection efficiency of the system; g_p is a dimensionless constant dependent on the shape of the laser pulse (0.664 for Gaussian and 0.558 for $sech^2$ pulse); PRF is the pulse repetition rate; and τ is the pulse duration in the sample plane. Some of these parameters can be altered to enhance the fluorescence. However, average excitation power is strictly limited by MPE, as discussed above, and cannot be exceeded. The pulse duration was kept as short as possible by incorporating dispersion compensation. An additional possibility for signal enhancement is the reduction of PRF. To verify this relationship, we have imaged *Abca4$^{-/-}$ Rdh8$^{-/-}$* mouse eyes at three different PRFs: 80, 20, and 4 MHz, as a function of average excitation power. **Supplementary Fig. 8** demonstrates that lower PRF allows generating more fluorescence photons at a given excitation average power. Each measurement series was fitted with an ax^b fitting function. A deviation from the theoretically expected function exponent of 2 can be explained by saturation of the photodetector and/or counting electronics. Lower PRF translates to a higher peak power of the excitation pulses, which generated more fluorescence photons per pulse. Thus, the more significant deviation for lower PRF.

Image processing. Eye movement causes a significant problem for obtaining high contrast TPEF images through averaging. It occurs not only between the acquisition of each frame but also within the collection of single-frame data. Hence, a global eye shift estimation is not

sufficient for a proper eye movement correction, and separate shifts for each recorded pixel in each frame compared to the reference frame have to be calculated. Then all frames have to be warped (digitally reshaped) to agree with the reference frame. For such alignment, we proposed a three-step procedure presented in Supplementary Information, Figure S7. In our method, we used reflectance images to track eye movement during TPEF acquisition. First, we subtracted the reflectance background from all reflectance frames (lens reflection), and we normalized the intensity through division by a mean intensity within the aperture. Next, we manually selected a single high-quality frame from the set of recorded reflectance images, which we later used as a reference frame. By "high-quality" we mean a frame without large visible vertical movement or eyelid presence that would cause missing data. In the following steps, we aligned other frames with the selected one. We applied bi-dimensional Empirical Mode Decomposition (EMD) to all reflectance frames to prepare data for this alignment. The bi-dimensional EMD is the extension of the one-dimensional EMD into a two-dimensional signal, and it decomposes the input signal into a few Intrinsic Mode Functions (IMF) and a residue (10). We used the Fast and Adaptive bi-dimensional EMD algorithm (11). The selection of a proper IMF allowed us to remove elements that do not follow eye movement; *e.g.*, aperture and the remaining part of the background. It also helped with noise filtration. The selected IMFs contained high-contrast images of blood vessels, which allow us to extract the displacement for each frame. Next, we calculated the transformations (transformation set) between consecutive frames' IMFs and the reference frame IMF in the following steps:

1. A fast XY shift calculation was performed through cross-correlation in Fourier space to have a better starting point for the iterative procedure.
2. The shift to the corrected IMF image was applied.
3. An iterative procedure was used to find affine transforms between the shifted IMF and reference IMF using the Matlab function "imregtform".
4. The transform to an already shifted IMF image was applied.

5. Iteratively, displacement fields were identified (x and y pixel shift map) between the transformed and reference IMFs using Matlab function "imregdemons".

After we obtained N-1 sets of parameters for three transformations (shift, affine, displacement field), we applied these sets to corresponding TPEF and reflectance images; *i.e.*, we warped the images. Then the quality of warped reflectance images inside aperture was verified through the calculation of mean-squared error. This check aimed to reject frames missing a large amount of data, such as closed eyelid or large vertical movement. Reference frames and frame pairs for which the quality check was passed were used for obtaining final reflectance and TPEF images.

Scotopic visual field test

A clinically-standard visual field test was supplemented by perimetry performed in scotopic conditions. The examination was done with a custom instrument using two types of stimuli that trigger two different mechanisms of visual perception: two-photon activation of visual pigments induced by pulsed near-IR laser (30), and one-photon (normal) activation induced by visible light. For this purpose, the system used two light sources: visible continuous-wave laser (λ_c = 514.2 nm, $\Delta\lambda_{FWHM}$ = 2.7 nm), and pulsed near-IR laser (λ_c = 1028.4 nm, $\Delta\lambda_{FWHM}$ = 27.0 nm, τ = 12.2 ps, PRF = 19.17 MHz). Both lasers shared the same optical path through the instrument to the subject's eye. Details of two-photon perimetry have been already published (13, 14). Briefly, the working principle is the following. During the test, the power of the stimulus was adjusted using a gradient filter steered by a stepper motor. The power level was continuously recorded by a power meter. Galvanometric scanners were used to form the desired shape of stimulus, and display it onto a specified retinal location. The subject's eye position was continuously monitored using a pupil camera, and modified by an operator if necessary. Moreover, the volunteer was asked to fix his/her gaze on a fixation point, a faint red dot in the center of the visual field, to help maintain the eye immobilized. The visual thresholds were determined using the 4-2-1 staircase method (13). The stimulus was a flicker circle with a diameter of 0.5 deg (Goldmann size III), periodically displayed onto the retina for 0.2 s every

0.8 s (0.25 duty cycle). During the tests, 25 or 17 retinal locations (up to ± 5 deg) were tested (13, 14).

The measurements were performed in a dark room (<0.01 lux). Before the first psychophysical test, the subject was dark-adapted for 25 min with eye patches on both eyes. The psychophysical procedure started with a near-IR laser. 10 min breaks between psychophysical tests with the visible and near-IR lasers were maintained to exclude the influence of a subject's tiredness on the test result. The pupil was dilated with 1% Tropicamide drops applied before the first adaptation period for the subject in the study shown in Figure 4. The control psychophysical tests after TPEF measurements were performed after a 15 min break, following the same conditions as before. During the experiments, all beam powers were maintained below the ANSI Z136.1:2014 limits. The method to determine the threshold of vision and two-photon sensitivity is described below.

The visual threshold (T_V) was assumed to be a geometric mean of those two power levels and is calculated using the following formula:

$$T_V = \sqrt{P_L \cdot P_H}$$

, where the test procedure enables us to find the highest power of the unseen stimulus (P_H) and the lowest power of the perceived stimulus (P_L).

The one- and two-photon sensitivities S are calculated the following way:

$$S[dB] = 10 \log \frac{T_{ref}}{T_V}$$

, where T_{ref} is a reference power level – 40 pW for visible stimulus and 400 µW for the near-IR stimulus. To demonstrate any possible change in visual field, the sensitivity values for a specific subject before and after TPEF imaging were averaged, and presented as the mean value ± standard deviation.

References

1. Trebino R, DeLong KW, Fittinghoff DN, Sweetser JN, Krumbugel MA, Richman BA, et al. Measuring ultrashort laser pulses in the time-frequency domain using frequency-resolved optical gating. *Rev Sci Instrum.* 1997;68(9):3277-95.
2. Zipfel WR, Williams RM, and Webb WW. Nonlinear magic: multiphoton microscopy in the biosciences. *Nat Biotechnol.* 2003;21(11):1368-76.

3. Kaschke M, Donnerhacke KH, and Rill MS. Optics of the Human Eye. *Optical Devices in Ophthalmology and Optometry: Technology, Design Principles, and Clinical Applications.* 2014:15-48.

4. Delori FC, Webb RH, and Sliney DH. Maximum permissible exposures for ocular safety (ANSI 2000), with emphasis on ophthalmic devices. *J Opt Soc Am A.* 2007;24(5):1250-65.

5. American National Standard for Safe Use of Lasers ANSI Z136.1. *Publisher Laser Institute of America.* 2014.

6. Bedggood P, Daaboul M, Ashman R, Smith G, and Metha A. Characteristics of the human isoplanatic patch and implications for adaptive optics retinal imaging. *J Biomed Opt.* 2008;13(2).

7. Morgan JI, Hunter JJ, Masella B, Wolfe R, Gray DC, Merigan WH, et al. Light-induced retinal changes observed with high-resolution autofluorescence imaging of the retinal pigment epithelium. *Invest Ophthalmol Vis Sci.* 2008;49(8):3715-29.

8. Masella BD, Williams DR, Fischer WS, Rossi EA, and Hunter JJ. Long-term reduction in infrared autofluorescence caused by infrared light below the maximum permissible exposure. *Invest Ophthalmol Vis Sci.* 2014;55(6):3929-38.

9. Schwarz C, Sharma R, Fischer WS, Chung MN, Palczewska G, Palczewski K, et al. Safety assessment in macaques of light exposures for functional two-photon ophthalmoscopy in humans. *Biomed Opt Express.* 2016;7(12):5148-69.

10. Huang NE, Shen Z, Long SR, Wu MLC, Shih HH, Zheng QN, et al. The empirical mode decomposition and the Hilbert spectrum for nonlinear and non-stationary time series analysis. P Roy Soc a-Math Phy. 1998;454(1971):903-95.

11. Wielgus M, Antoniewicz A, Bartyś M, and Putz B. 2012 15th International Conference on Information Fusion. IEEE; 2012:649-54.

12. Palczewska G, Vinberg F, Stremplewski P, Bircher MP, Salom D, Komar K, et al. Human infrared vision is triggered by two-photon chromophore isomerization. P Natl Acad Sci USA. 2014;111(50):E5445-E54.

13. Ruminski D, Palczewska G, Nowakowski M, Zielinska A, Kefalov VJ, Komar K, et al. Two-photon microperimetry: sensitivity of human photoreceptors to infrared light. Biomed Opt Express. 2019;10(9):4551-67.

14. Marzejon MJ, Kornaszewski L, Boguslawski J, Ciacka P, Martynow M, Palczewska G, et al. Two-photon microperimetry with picosecond pulses. Biomed Opt Express. 2021;12(1):462-79.
